# Supplementary material for: Mechanistic Elucidation of the Anti-Ageing Effects of Dendrobium officinale via Network Pharmacology and Experimental Validation
Source: Foods. 2025 Oct 3;14(19):3418. doi: 10.3390/foods14193418 (PMC12523731; doi:10.3390/foods14193418)
Supplement: Supplementary file 1 [file foods-14-03418-s001.zip › Supplementary_Material..pdf]

Table S1 Metabolite Profiling (370 Compounds) and Anti-aging Target Network Prediction in *Dendrobium officinale* Extracts

ID	m/z	Retention time (min)	Metabolites	Canonical SMILES	Compound ID	Super Class	Class	Formula	Mass Error (ppm)
0.81_549.1673m/z	549.167	0.810	Gentiotriose	C(C1C(C(C(C(O1)OCC2C(C(C(C(O2)OCC3C(C(C(C(O3)O)O)O)O)O)O)O)O)O)O)O)O	HMDB0029910	Organic oxygen compounds	Organooxygen compounds	C18H32O16	0.132
4.93_493.2291m/z	493.229	4.926	Acuminoside	CC(=CCCC(=CCOC1C(C(C(C(O1)COC2C(C(CO2)(CO)O)O)O)O)O)O)C)C	HMDB0029347	Lipids and lipid-like molecules	Prenol lipids	C21H36O10	0.009
3.95_547.1457m/z	547.146	3.952	Cassiaside B	CC1=CC(=O)C2=C(C3=C(C=C(C=C3C=C2O1)OC)OC4C(C(C(C(O4)COC5C(C(CO5)(CO)O)O)O)O)O)O	HMDB0038477	Organoheterocyclic compounds	Naphthopyrans	C26H30O14	-0.018
5.86_273.0756m/z	273.076	5.855	Naringenin		HMDB0002670	Phenylpropanoids and polyketides	Flavonoids	C15H12O5	-0.543
14.34_566.4132n	567.420	14.345	Nigroxanthin	C\C(\C=C\C=C(/C)\C=C\C1=C(C)CC(O)CC1(C)C)=C/C=C/C=C(\C)/C=C/C=C(\C)/C=C/C1(O)C(=C)C=CCC1(C)C	HMDB0037122	Lipids and lipid-like molecules	Prenol lipids	C40H54O2	1.368
0.82_265.1161n	266.123	0.816	D-1-[(3-Carboxypropyl)amino]-1-deoxyfructose	C(CC(=O)O)CNCC1(C(C(C(O1)CO)O)O)O	HMDB0038663	Organic acids and derivatives	Carboxylic acids and derivatives	C10H19NO7	-0.253

3.80_578.1635n	577.156	3.803	Chrysophanol 8-gentiobioside	CC1=CC2=C(C(=C1)O)C(=O)C3=C(C2=O)C=CC=C3OC4C(C(C(C(O4)COC5C(C(C(C(O5)CO)O)O)O)O)O)O	HMDB0039488	Benzenoids	Anthracenes	C27H30O14	-0.056
8.81_309.2072m/z	309.207	8.814	Corchorifatty acid F	CCC=CCC(C(C=CC(CCCCCCC(=O)O)O)O)O	HMDB0035919	Lipids and lipid-like molecules	Fatty Acyls	C18H32O5	0.282
4.06_631.1282m/z	631.128	4.059	6''-Malonylapiin	C1C(C(C(O1)OC2C(C(C(OC2OC3=CC(=C4C(=C3)OC(=CC4=O)C5=CC=C(C=C5)O)O)COC(=O)CC(=O)O)O)O)O)(CO)O	HMDB0037601	Phenylpropanoids and polyketides	Flavonoids	C29H30O17	-3.470
9.31_279.2317m/z	279.232	9.311	Avenoleic acid		HMDB0029978	Lipids and lipid-like molecules	Fatty Acyls	C18H32O3	-0.475
8.25_302.3052m/z	302.305	8.246	Sphinganine	CCCCCCCCCCCCCCCC(C(CO)N)O	HMDB0000269	Organic nitrogen compounds	Organonitrogen compounds	C18H39NO2	-0.505
4.07_565.1549m/z	565.155	4.067	Stachyoside A	CC1(CC(C2(C1C(OC=C2)OC3C(C(C(C(O3)COC4C(C(C(C(O4)CO)O)O)O)O)O)O)O)O)O	HMDB0039092	Lipids and lipid-like molecules	Prenol lipids	C21H34O15	3.809
0.82_234.0971m/z	234.097	0.816	N-(1-Deoxy-1-fructosyl)alanine	CC(C(=O)O)NCC1(C(C(C(O1)CO)O)O)O	HMDB0038662	Organic oxygen compounds	Organooxygen compounds	C9H17NO7	-0.433

3.52_612.1691n	593.151	3.524	Aromadendrin 3,7-diglucoside	C1=CC(=CC=C1C2C(C(=O)C3=C(C=C(C=C3O2)OC4C(C(C(O4)CO)O)O)O)O)OC5C(C(C(C(O5)CO)O)O)O)O	HMDB0040559	Phenylpropanoids and polyketides	Flavonoids	C27H32O16	0.184	
3.76_613.2139m/z	613.214	3.760	Citrusin B	COC1=CC(=CC(=C1OC(CO)C(C2=CC(=C(C=C2)OC3C(C(C(C(O3)CO)O)O)O)OC)O)OC)C=CCO	HMDB0039231	Lignans, neolignans and related compounds	Lignan glycosides	C27H36O13	0.238	8.75
3.70_563.1406m/z	563.141	3.696	Kaempferol 3-arabinofuranoside 7-rhamnofuranoside	CC(C1C(C(C(O1)OC2=CC(=C3C(=C2)OC(=C(C3=O)OC4C(C(C(O4)CO)O)O)C5=CC=C(C=C5)O)O)O)O)O	HMDB0037575	Phenylpropanoids and polyketides	Flavonoids	C26H28O14	-0.075	
5.15_288.0635n	287.056	5.149	Norartocarpanone		HMDB0037314	Phenylpropanoids and polyketides	Flavonoids	C15H12O6	0.305	
4.10_799.2096m/z	799.210	4.103	Spinosin C	COC1=C(C(=C2C(=C1)OC(=CC2=O)C3=CC=C(C=C3)O)O)C4C(C(C(C(O4)CO)O)O)OC5C(C(C(C(O5)COC(=O)C=CC6=CC=C(C=C6)O)O)O)O	HMDB0037463	Phenylpropanoids and polyketides	Flavonoids	C37H38O17	0.650	
7.82_431.2651m/z	431.265	7.825	Mangalkanyl glucoside	CCCCCCCCCC1CCC=CC1OC2C(C(C(C(O2)CO)O)O)O	HMDB0036015	Organic oxygen compounds	Organooxygen compounds	C21H38O6	0.239	

3.80_578.1632n	579.170	3.797	Acacetin 7-[apiosyl(1->6)-glucoside]	COC1=CC=C(C=C1)C2=CC(=O)C3=C(C=C(C=C3O2)OC4C(C(C(C(O4)COC5C(C(CO5)(CO)O)O)O)O)O)O	HMDB0035023	Phenylpropanoids and polyketides	Flavonoids	C27H30O14	-0.601
4.24_387.1436m/z	387.144	4.236	Pinostilbenoside	COC1=CC(=CC(=C1)O)C=CC2=CC=C(C=C2)OC3C(C(C(C(O3)CO)O)O)O	HMDB0039273	Phenylpropanoids and polyketides	Stilbenes	C21H24O8	-0.690
0.83_116.0708m/z	116.071	0.833	L-Proline		HMDB0000162	Organic acids and derivatives	Carboxylic acids and derivatives	C5H9NO2	2.036
3.76_593.1513m/z	593.151	3.760	Apigenin 7-[galactosyl-(1->4)-mannoside]	C1=CC(=CC=C1C2=CC(=O)C3=C(C=C(C=C3O2)OC4C(C(C(C(O4)CO)OC5C(C(C(C(O5)CO)O)O)O)O)O)O	HMDB0037852	Phenylpropanoids and polyketides	Flavonoids	C27H30O15	0.254
3.98_575.2100m/z	575.210	3.983	Osmanthuside B	CC1C(C(C(C(O1)OC2C(C(OC(C2OC(=O)C=CC3=CC=C(C=C3)O)CO)OCCC4=CC=C(C=C4)O)O)O)O	HMDB0038749	Phenylpropanoids and polyketides	Cinnamic acids and derivatives	C29H36O13	-3.855
3.85_669.1678m/z	669.168	3.845	6,8-Diglucosyldiosmetin	COC1=C(C=C(C=C1)C2=CC(=O)C3=C(C(=C(C(=C3O2)C4C(C(C(C(O4)CO)O)O)O)O)C5C(C(C(C(O5)CO)O)O)O)O)O	HMDB0037410	Phenylpropanoids and polyketides	Flavonoids	C28H32O16	0.960
8.31_460.2691m/z	460.269	8.310	Pectachol	CC1(C2CCC(=C)C(C2(CCC1O)C)COC3=C(C=C4C=CC(=O)OC4=C3OC)OC)C	HMDB0039064	Phenylpropanoids and polyketides	Coumarins and derivatives	C26H34O6	-0.538

3.53_594.1587n	595.166	3.528	Graveobioside B	COC1=C(C=CC(=C1)C2=CC(=O)C3=C(C=C(C=C3O2)OC4C(C(C(C(O4)CO)O)O)OC5C(C(CO5)(CO)O)O)O)O	HMDB0037454	Phenylpropanoids and polyketides	Flavonoids	C27H30O15	0.379
3.87_609.1463m/z	609.146	3.867	Rutin	CC1C(C(C(C(O1)OCC2C(C(C(C(O2)OC3=C(OC4=CC(=CC(=C4C3=O)O)O)C5=CC(=C(C=C5)O)O)O)O)O)O)O)O	HMDB0003249	Phenylpropanoids and polyketides	Flavonoids	C27H30O16	0.374
3.70_435.2242m/z	435.224	3.696	(3S,5R,6S,7E,9x)-7-Megastigmene-3,6,9-triol 9-glucoside		HMDB0041176	Lipids and lipid-like molecules	Fatty Acyls	C19H34O8	1.485
0.86_337.0777m/z	337.078	0.860	2-O-beta-D-Glucopyranuronosyl-D-mannose		HMDB0039722	Organic oxygen compounds	Organooxygen compounds	C12H20O12	0.249
3.71_564.1477n	565.155	3.714	Apigenin 6-C-glucoside 8-C-arabinoside	C1=CC(=CC=C1C2=CC(=O)C3=C(C(=C(C(=C3O2)C4C(C(C(O4)CO)O)O)O)O)C5C(C(C(C(O5)CO)O)O)O)O)O	HMDB0029260	Phenylpropanoids and polyketides	Flavonoids	C26H28O14	-0.303
4.21_769.1979m/z	769.198	4.211	Isovitexin 2''-O-(6'''-feruloyl)glucoside	COC1=C(C=CC(=C1)C=CC(=O)OCC2C(C(C(C(O2)OC3C(C(C(OC3C4=C(C5=C(C=C4O)OC(=CC5=O)C6=CC=C(C=C6)O)O)CO)O)O)O)O)O)O	HMDB0038042	Phenylpropanoids and polyketides	Flavonoids	C37H38O18	-0.809

8.56_263.2368m/z	263.237	8.565	Mangiferic acid	CCC=CCCCCC=CCCCCCCCC(=O)O	HMDB0029800	Lipids and lipid-like molecules	Fatty Acyls	C18H32O2	-0.394
3.55_641.1723m/z	641.172	3.546	(2R)-6,8-Diglucopyranosyl-4',5,7-trihydroxyflavanoneC1C(OC2=C(C(=C(C(=C2C1=O)O)C3C(C(C(C(O3)CO)O)O)O)O)C4C(C(C(C(O4)CO)O)O)O)C5=CC=C(C=C5)O		HMDB0037407	Phenylpropanoids and polyketides	Flavonoids	C27H32O15	-0.025
4.32_372.2146n	395.204	4.321	Blumenol C glucoside	CC1=CC(=O)CC(C1CCC(C)OC2C(C(C(C(O2)CO)O)O)O)(C)C	HMDB0040668	Lipids and lipid-like molecules	Fatty Acyls	C19H32O7	-0.435
3.57_583.2034m/z	583.203	3.567	Citrusin A	COC1=C(C=CC(=C1)C=CCO)OC(CO)C(C2=CC(=C(C=C2)OC3C(C(C(C(O3)CO)O)O)O)OC)O	HMDB0039230	Lignans, neolignans and related compounds	Lignan glycosides	C26H34O12	0.353
4.24_675.2625m/z	675.262	4.236	Crocin 3	CC(=CC=CC=C(C)C=CC=C(C)C(=O)OC1C(C(C(C(O1)COC2C(C(C(C(O2)CO)O)O)O)O)O)O)C=CC=C(C)C(=O)O	HMDB0039121	Lipids and lipid-like molecules	Prenol lipids	C32H44O14	0.218
8.80_277.2161m/z	277.216	8.799	Stearidonic acid		HMDB0006547	Lipids and lipid-like molecules	Fatty Acyls	C18H28O2	-0.463

3.55_385.1140m/z	385.114	3.546	Oleoside 11-methyl ester		HMDB0041550	Lipids and lipid-like molecules	Prenol lipids	C17H24O11	0.031
14.56_463.3781m/z	463.378	14.558	(3beta,22R,23R,24S)-3,22,23-Trihydroxystigmastan-6-one	CCC(C(C)C)C(C(C(C)C1CCC2C1(CCC3C2CC(=O)C4C3(CCC(C4)O)C)C)O)O	HMDB0039713	Lipids and lipid-like molecules	Steroids and steroid derivatives	C29H50O4	-0.249
3.70_549.2550m/z	549.255	3.696	(3S,7E,9R)-4,7-Megastigmadiene-3,9-diol 9-[apiosyl-(1->6)-glucoside]	CC1=CC(CC(C1C=CC(C)OC2C(C(C(C(O2)COC3C(C(CO3)(CO)O)O)O)O)O)(C)C)O	HMDB0029766	Lipids and lipid-like molecules	Fatty Acyls	C24H40O11	-0.543
4.08_605.2816m/z	605.282	4.081	N5-Acetyl-N2-gamma-L-glutamyl-L-ornithine		HMDB0039423	Organic acids and derivatives	Carboxylic acids and derivatives	C12H21N3O6	4.633
13.33_292.2765n	310.310	13.331	Geranylcitronellol	CC(CCC=C(C)CCC=C(C)CCC=C(C)C)CCO	HMDB0032147	Lipids and lipid-like molecules	Prenol lipids	C20H36O	-0.460
8.41_313.2385m/z	313.238	8.414	9,10-DHOME	CCCCCC=CCC(C(CCCCCCCC(=O)O)O)O	HMDB0004704	Lipids and lipid-like molecules	Fatty Acyls	C18H34O4	0.209
5.15_193.0500m/z	193.050	5.149	Isoferulic acid	COC1=C(C=C(C=C1)C=CC(=O)O)O	HMDB0000955	Phenylpropanoids and polyketides	Cinnamic acids and derivatives	C10H10O4	-3.385

8.32_676.3672n	721.366	8.316	(S)-Nerolidol 3-O-[a-L-Rhamnopyranosyl-(1->4)-a-L-rhamnopyranosyl-(1->2)-b-D-glucopyranoside]	CC1C(C(C(C(O1)OC2C(OC(C(C2O)O)OC3C(C(C(OC3OC(C)(CCC=C(C)CCC=C(C)C)C=C)CO)O)O)C)O)O)O	HMDB0040845	Organic oxygen compounds	Organooxygen compounds	C33H56O14	0.307
3.82_509.2238m/z	509.224	3.824	Linalool oxide D 3-[apiosyl-(1->6)-glucoside]	CC1(C(CCC(O1)(C)C=C)OC2C(C(C(C(O2)COC3C(C(CO3)(CO)O)O)O)O)O)C	HMDB0031367	Organic oxygen compounds	Organooxygen compounds	C21H36O11	-0.325
0.98_565.0476m/z	565.048	0.979	Uridine diphosphate glucose	C1=CN(C(=O)NC1=O)C2C(C(C(O2)COP(=O)(O)OP(=O)(O)OC3C(C(C(C(O3)CO)O)O)O)O)O	HMDB0000286	Nucleosides, nucleotides, and analogues	Pyrimidine nucleotides	C15H24N2O17P2	-0.275
1.00_527.1579m/z	527.158	1.004	beta-D-Galactopyranosyl-(1->3)-beta-D-galactopyranosyl-(1->6)-D-galactose	C(C1C(C(C(C(O1)OC2C(C(OC(C2O)OCC3C(C(C(C(O3)O)O)O)O)CO)O)O)O)O)O	HMDB0038853	Organic oxygen compounds	Organooxygen compounds	C18H32O16	-0.638
4.21_269.1746m/z	269.175	4.215	4,11,13,15-Tetrahydroridentin B		HMDB0036150	Lipids and lipid-like molecules	Prenol lipids	C15H24O4	-0.582
4.47_475.2186m/z	475.219	4.470	Ustiloxin D		HMDB0041054	Organic acids and derivatives	Carboxylic acids and derivatives	C23H34N4O8	-2.495

3.94_341.1382m/z	341.138	3.941	(-)-Matairesinol		HMDB0035698	Lignans, neolignans and related compounds	Furanoid lignans	C20H22O6	-0.296
4.34_739.1883m/z	739.188	4.341	Kaempferol 3-[2''-(p-coumaroylglucosyl)rhamnoside]	CC1C(C(C(C(O1)OC2=C(OC3=CC(=CC(=C3C2=O)O)O)C4=CC=C(C=C4)O)OC5C(C(C(C(O5)COC(=O)C=CC6=CC=C(C=C6)O)O)O)O)O)O	HMDB0040538	Phenylpropanoids and polyketides	Flavonoids	C36H36O17	0.415
5.64_607.2149m/z	607.215	5.642	Musabalbisiane C	CC=C(C)C(=O)OC1CCC2(C3(CC(OC3O)C4=COC=C4)C(CC(C2(C1(CC(=O)O)CO)CO)O)CO)CO	HMDB0038682	Lipids and lipid-like molecules	Prenol lipids	C28H40O12	-0.345
8.44_296.2350n	297.242	8.437	12-Hydroxy-8,10-octadecadienoic acid		HMDB0029998	Lipids and lipid-like molecules	Fatty Acyls	C18H32O3	-0.426
4.96_527.2466m/z	527.247	4.959	Cinncassiol A 19-glucoside	CC1CCC2(C3(CC(=O)OC2(C1O)C4(C3(CC(=C4C)C(C)COC5C(C(C(C(O5)CO)O)O)O)O)O)C)O	HMDB0035165	Lipids and lipid-like molecules	Prenol lipids	C26H40O12	-3.789
3.90_135.1169m/z	135.117	3.901	(1S,4S)-Dihydrocarvone		HMDB0036080	Lipids and lipid-like	Prenol lipids	C10H16O	0.256

						molecules			
2.29_148.0525n	166.086	2.289	3,4-Dihydro-2H-1-benzopyran-2-one		HMDB0036626	Phenylpropanoids and polyketides	3,4-dihydrocoumarins	C9H8O2	0.510
0.82_432.1709m/z	432.171	0.816	a-L-Arabinofuranosyl-(1->3)-[a-L-arabinofuranosyl-(1r5)]-L-arabinose		HMDB0041223	Organic oxygen compounds	Organooxygen compounds	C15H26O13	-0.527
3.95_827.2986m/z	827.299	3.952	Ptelatoside A		HMDB0032600	Organic oxygen compounds	Organooxygen compounds	C19H26O10	0.795
3.93_567.2080m/z	567.208	3.931	Isolariciresinol 4'-O-beta-D-glucoside	COC1=C(C=C2C(C(C(CC2=C1)CO)CO)C3=CC(=C(C=C3)OC4C(C(C(C(O4)CO)O)O)O)OC)O	HMDB0040471	Lignans, neolignans and related compounds	Lignan glycosides	C26H34O11	-0.653
9.71_277.2161m/z	277.216	9.709	4,8,12,15-Octadecatetraenoic acid		HMDB0032672	Lipids and lipid-like molecules	Fatty Acyls	C18H28O2	-0.394
3.42_461.1667m/z	461.167	3.417	Verbasoside	CC1C(C(C(C(O1)OC2C(C(OC(C2O)OCCC3=CC(=C(C=C3)O)O)CO)O)O)O)O	HMDB0039233	Organic oxygen compounds	Organooxygen compounds	C20H30O12	0.505

4.28_551.2116m/z	551.212	4.278	Tracheloside	COC1=C(C=C(C=C1)CC2COC(=O)C2(CC3=CC(=C(C=C3)OC4C(C(C(C(O4)CO)O)O)O)OC)O)OC	HMDB0030557	Lignans, neolignans and related compounds	Lignan glycosides	C27H34O12	-1.262
3.78_595.1656m/z	595.166	3.777	Hydroxysafflor yellow A	C1=CC(=CC=C1C=CC(=C2C(=C(C(=O)C(C2=O)(C3C(C(C(C(O3)CO)O)O)O)O)C4C(C(C(C(O4)CO)O)O)O)O)O)O	HMDB0040677	Phenylpropanoids and polyketides	Cinnamic acids and derivatives	C27H32O16	-0.237
0.80_357.1038m/z	357.104	0.804	4-O-beta-D-Galactopyranosyl-D-xylose		HMDB0038864	Organic oxygen compounds	Organooxygen compounds	C11H20O10	-0.047
3.40_373.1832m/z	373.183	3.403	trans-p-Menthane-1,7,8-triol 8-glucoside		HMDB0034784	Organic oxygen compounds	Organooxygen compounds	C16H30O8	-0.292
4.34_461.2395m/z	461.240	4.341	Citronellyl beta-sophoroside	CC(CCC=C(C)C)CCOC1C(C(C(C(O1)CO)O)O)OC2C(C(C(C(O2)CO)O)O)O	HMDB0032839	Lipids and lipid-like molecules	Prenol lipids	C22H40O11	0.608
5.34_341.1969m/z	341.197	5.341	Tanacetol B		HMDB0035075	Lipids and lipid-like molecules	Prenol lipids	C17H28O4	-0.075

3.52_477.1614m/z	477.161	3.524	Kelampayoside A	COC1=CC(=CC(=C1OC)OC)OC2C(C(C(C(O2)COC3C(C(CO3)(CO)O)O)O)O)O	HMDB0038714	Organic oxygen compounds	Organooxygen compounds	C20H30O13	0.151
7.93_293.2110m/z	293.211	7.927	[7]-Paradol		HMDB0040806	Benzenoids	Phenols	C18H28O3	-0.493
4.21_458.1417n	481.132	4.215	cis-p-Coumaric acid 4-[apiosyl-(1->2)-glucoside]		HMDB0037088	Organic oxygen compounds	Organooxygen compounds	C20H26O12	-1.567
4.90_383.1347m/z	383.135	4.904	Benzyl O-[arabinofuranosyl-(1->6)-glucoside]		HMDB0041514	Organic oxygen compounds	Organooxygen compounds	C18H26O10	-0.025
7.34_271.0964m/z	271.096	7.341	4-O-Methylpinosylvic acid		HMDB0040868	Phenylpropanoids and polyketides	Stilbenes	C16H14O4	-0.218
3.65_371.1490m/z	371.149	3.652	Isoxanthohumol B		HMDB0035499	Phenylpropanoids and polyketides	Linear 1,3-diarylpropanoids	C21H22O6	0.278
4.88_193.0863m/z	193.086	4.882	Zingerone		HMDB0032590	Benzenoids	Phenols	C11H14O3	-3.564
2.74_405.1402m/z	405.140	2.742	2'-Methoxy-3-(2,4-dihydroxyphenyl)-1,2-propanediol 4'-glucoside		HMDB0039473	Organic oxygen compounds	Organooxygen compounds	C16H24O9	-0.119
4.13_235.1692m/z	235.169	4.131	6alpha-Carissanol		HMDB0035309	Lipids and lipid-like molecules	Prenol lipids	C15H24O3	-0.311

14.34_600.4177n	583.414	14.345	Capsanthin 3,6-epoxide	CC(=CC=CC=C(C)C=CC=C(C)C=CC12C(CC(O1)CC2(C)O)(C)C)C=CC=C(C)C=CC(=O)C3(CC(CC3(C)C)O)C	HMDB0033260	Lipids and lipid-like molecules	Prenol lipids	C40H56O4	-0.246
3.67_386.1940n	431.192	3.674	Corchoionol C 9-glucoside		HMDB0029772	Lipids and lipid-like molecules	Fatty Acyls	C19H30O8	-0.218
3.08_625.2102m/z	625.210	3.082	Acteoside	CC1C(C(C(C(O1)OC2C(C(OC(C2OC(=O)C=CC3=CC(=C(C=C3)O)O)CO)OCCC4=CC(=C(C=C4)O)O)O)O)O)O	HMDB0034843	Phenylpropanoids and polyketides	Cinnamic acids and derivatives	C29H36O15	-4.056
3.57_250.1568n	251.164	3.569	(6beta,8alpha)-6-Hydroxy-7(11)-eremophilen-12,8-olide		HMDB0035148	Lipids and lipid-like molecules	Prenol lipids	C15H22O3	-0.400
3.89_307.0820m/z	307.082	3.888	trans-o-Coumaric acid 2-glucoside		HMDB0033581	Organic oxygen compounds	Organooxygen compounds	C15H18O8	-1.047
0.87_118.0865m/z	118.086	0.867	L-Valine		HMDB0000883	Organic acids and derivatives	Carboxylic acids and derivatives	C5H11NO2	1.797
4.62_559.2760m/z	559.276	4.622	Capsianoside V	CC(=CCCC(=CCCC(C)(C=C)OC1C(C(C(C(O1)CO)O)O)O)CO)CC(C=C(C)C(=O)O)O	HMDB0030737	Lipids and lipid-like molecules	Prenol lipids	C26H42O10	-0.080

4.06_563.1405m/z	563.140	4.059	Kaempferol 3-rhamnoside 7-xyloside		HMDB0039319	Phenylpropanoids and polyketides	Flavonoids	C26H28O14	-0.241
0.80_146.0692n	147.076	0.799	L-Glutamine		HMDB0000641	Organic acids and derivatives	Carboxylic acids and derivatives	C5H10N2O3	0.654
4.28_249.1484m/z	249.148	4.278	Artemin		HMDB0034696	Lipids and lipid-like molecules	Prenol lipids	C15H22O4	-0.308
4.99_503.2501m/z	503.250	4.990	[6]-Gingerdiol 5-O-beta-D-glucopyranoside	CCCCCC(CC(CCC1=CC(=C(C=C1)O)OC)O)OC2C(C(C(C(O2)CO)O)O)O	HMDB0036123	Lipids and lipid-like molecules	Fatty Acyls	C23H38O9	0.634
3.86_610.1529n	611.160	3.859	Quercetin 7-(rhamnosylglucoside)	CC1C(C(C(C(O1)OC2C(C(C(OC2OC3=CC(=C4C(=C3)OC(=C(C4=O)O)C5=CC(=C(C=C5)O)O)O)CO)O)O)O)O)O	HMDB0039383	Benzenoids	Benzene and substituted derivatives	C27H30O16	-0.754
1.18_129.0427n	130.050	1.184	Pyroglutamic acid		HMDB0000267	Organic acids and derivatives	Carboxylic acids and derivatives	C5H7NO3	0.746
6.26_453.1556m/z	453.156	6.259	Mammea A/AC cyclo F		HMDB0037242	Phenylpropanoids and polyketides	Neoflavonoids	C24H24O6	0.321
3.44_348.1298m/z	348.130	3.438	Casuarine 6-alpha-D-glucoside		HMDB0031999	Organic oxygen compounds	Organooxygen compounds	C14H25NO10	-0.687

4.12_459.2237m/z	459.224	4.125	(4R,5S,7R,11S)-11,12-Dihydroxy-1(10)-spirovetiven-2-one 11-glucoside	CC1CC(=O)C=C(C12CCC(C2)C(C)(CO)OC3C(C(C(C(O3)CO)O)O)O)C	HMDB0033150	Lipids and lipid-like molecules	Prenol lipids	C21H34O8	0.371
4.30_207.0652m/z	207.065	4.300	Citropten		HMDB0032952	Phenylpropanoids and polyketides	Coumarins and derivatives	C11H10O4	0.139
0.72_156.0768m/z	156.077	0.721	L-2-Amino-3-(1-pyrazolyl)propanoic acid		HMDB0034267	Organic acids and derivatives	Carboxylic acids and derivatives	C6H9N3O2	0.283
0.87_180.1019m/z	180.102	0.867	(R)-Salsolinol		HMDB0005199	Organoheterocyclic compounds	Tetrahydroisoquinolines	C10H13NO2	-0.073
0.87_191.0190m/z	191.019	0.865	Isocitric acid		HMDB0000193	Organic acids and derivatives	Carboxylic acids and derivatives	C6H8O7	-3.937
4.60_195.1016m/z	195.102	4.596	Methoxyeugenol		HMDB0041194	Benzenoids	Phenols	C11H14O3	0.084
3.65_351.1053m/z	351.105	3.652	Dihydromelilotoside		HMDB0038334	Organic oxygen compounds	Organooxygen compounds	C15H20O8	0.828
3.17_366.1429n	367.150	3.166	Tetrahydropentoxyline		HMDB0029992	Alkaloids and derivatives	Harmala alkaloids	C17H22N2O7	0.572
3.78_208.0735n	191.070	3.777	3-(3,4-Dimethoxyphenyl)-2-propenoic acid		HMDB0034315	Phenylpropanoids and polyketides	Cinnamic acids and derivatives	C11H12O4	-0.126
3.52_613.1781m/z	613.178	3.524	cis-Mulberroside A	C1=CC(=C(C=C1OC2C(C(C(C(O2)CO)O)O)O)O)C=CC3=CC(=CC(=C3)OC4C(C(C(C(O4)CO)O)O)O)O	HMDB0031726	Phenylpropanoids and polyketides	Stilbenes	C26H32O14	1.196

4.19_421.1470m/z	421.147	4.194	Methyl 3,4-dihydroxy-5-prenyl benzoate 3-glucoside		HMDB0040121	Phenylpropanoids and polyketides	Tannins	C19H26O9	0.340
3.49_397.1108m/z	397.111	3.486	Geniposidic acid		HMDB0034942	Lipids and lipid-like molecules	Prenol lipids	C16H22O10	0.764
3.87_623.1618m/z	623.162	3.867	Rhoifolin	CC1C(C(C(C(O1)OC2C(C(C(OC2OC3=CC(=C4C(=C3)OC(=CC4=O)C5=CC=C(C=C5)O)O)CO)O)O)O)O)O	HMDB0038848	Phenylpropanoids and polyketides	Flavonoids	C27H30O14	0.146
1.27_230.1023m/z	230.102	1.268	Linamarin		HMDB0033699	Organic oxygen compounds	Organooxygen compounds	C10H17NO6	-0.040
4.12_713.2668m/z	713.267	4.125	Isolimonic acid glucoside	CC1(C2CC(=O)C3(C(C2(CO1)C(CC(=O)O)O)CCC(C34C(O4)C(=O)O)(C)C(C5=COC=C5)OC6C(C(C(C(O6)CO)O)O)O)C)C	HMDB0039365	Lipids and lipid-like molecules	Saccharolipids	C32H44O15	0.929
4.11_311.1276m/z	311.128	4.109	Artocarbene		HMDB0031744	Phenylpropanoids and polyketides	Stilbenes	C19H18O4	-0.450
0.82_305.1346m/z	305.135	0.816	N2-Galacturonyl-L-lysine		HMDB0033105	Organic acids and derivatives	Carboxylic acids and derivatives	C12H22N2O8	0.772
3.94_253.1797m/z	253.180	3.941	Epioxylubimin		HMDB0035613	Lipids and lipid-like	Prenol lipids	C15H24O3	-0.500

						molecules			
0.85_204.1110n	205.118	0.850	N6-Acetyl-5S-hydroxy-L-lysine		HMDB0033891	Organic acids and derivatives	Carboxylic acids and derivatives	C8H16N2O4	0.002
1.20_191.0189m/z	191.019	1.197	D-threo-Isocitric acid		HMDB0001874	Organic acids and derivatives	Carboxylic acids and derivatives	C6H8O7	-4.448
4.41_460.1583n	459.151	4.405	Methyl salicylate O-[rhamnosyl-(1->6)-glucoside]		HMDB0033138	Organic oxygen compounds	Organooxygen compounds	C20H28O12	0.548
4.00_237.1848m/z	237.185	4.004	Aubergenone		HMDB0035827	Lipids and lipid-like molecules	Prenol lipids	C15H24O2	-0.398
13.40_508.3763n	531.366	13.395	Fasciculol C	CC1(C2CCC3=C(C2(CC(C1O)O)C)CC(C4(C3(CCC4C(CCC(C(C)(C)O)O)CO)C)C)O)C	HMDB0035853	Lipids and lipid-like molecules	Prenol lipids	C30H52O6	-0.123
0.82_120.0657m/z	120.066	0.816	L-Threonine		HMDB0000167	Organic acids and derivatives	Carboxylic acids and derivatives	C4H9NO3	1.529
4.12_457.2081m/z	457.208	4.125	Abscisic alcohol 11-glucoside		HMDB0039636	Lipids and lipid-like molecules	Prenol lipids	C21H32O8	0.435
5.41_261.1118m/z	261.112	5.408	2beta,9xi-Dihydroxy-8-oxo-1(10),4,11(13)-germacratrien-12,6alpha-olide		HMDB0036662	Lipids and lipid-like molecules	Prenol lipids	C15H18O5	-1.284

0.80_342.1162n	387.114	0.804	Trehalose		HMDB0000975	Organic oxygen compounds	Organooxygen compounds	C12H22O11	-0.001
5.85_331.0824m/z	331.082	5.847	3,4-Dihydroxy-9-methoxypterocarpan		HMDB0034012	Phenylpropanoids and polyketides	Isoflavonoids	C16H14O5	0.216
3.78_639.1541m/z	639.154	3.782	Scoparin 2''-xyloside	COC1=C(C=CC(=C1)C2=CC(=O)C3=C(O2)C(=C(C=C3O)O)C4C(C(C(C(O4)CO)O)O)OC5C(C(C(CO5)O)O)O)O	HMDB0038814	Phenylpropanoids and polyketides	Flavonoids	C27H30O15	-4.332
3.67_175.1482m/z	175.148	3.673	5,7alpha-Dihydro-1,4,4,7a-tetramethyl-4H-indene		HMDB0036683	Hydrocarbons	Unsaturated hydrocarbons	C13H18	0.191
4.02_251.0790n	232.061	4.016	N-Carboxyacetyl-D-phenylalanine		HMDB0039102	Organic acids and derivatives	Carboxylic acids and derivatives	C12H13NO5	-1.319
3.67_224.1413n	207.138	3.673	13-Oxo-9,11-tridecadienoic acid		HMDB0034564	Lipids and lipid-like molecules	Fatty Acyls	C13H20O3	0.200
3.42_326.0997n	349.090	3.424	trans-p-Coumaric acid 4-glucoside		HMDB0039509	Organic oxygen compounds	Organooxygen compounds	C15H18O8	-1.353

4.36_843.2678m/z	843.268	4.363	Foeniculoside I	C1=CC(=CC=C1C=CC2=C3C(C(OC3=CC(=C2)O)C4=CC=C(C=C4)O)C5=C6C(C(OC6=CC(=C5)O)C7=CC=C(C=C7)O)C8=CC(=CC(=C8)OC9C(C(C(C(O9)CO)O)O)O)O)O	HMDB0041556	Phenylpropanoids and polyketides	2-arylbenzofuran flavonoids	C48H42O14	3.689
4.21_233.1535m/z	233.154	4.215	Lactaronecatorin A		HMDB0037529	Lipids and lipid-like molecules	Prenol lipids	C15H22O3	-0.365
3.85_463.2187m/z	463.219	3.845	Linalool 3,6-oxide primeveroside	CC1(CCC(O1)C(C)(C)OC2C(C(C(C(O2)COC3C(C(C(CO3)O)O)O)O)O)O)C=C	HMDB0035489	Organic oxygen compounds	Organooxygen compounds	C21H36O11	0.408
3.61_481.1318m/z	481.132	3.610	2'-(E)-Feruloyl-3-(arabinosylxylose)		HMDB0030230	Phenylpropanoids and polyketides	Cinnamic acids and derivatives	C20H26O12	0.327
0.83_179.0793n	162.076	0.833	Glucosamine		HMDB0001514	Organic oxygen compounds	Organooxygen compounds	C6H13NO5	-0.193
5.86_272.0684n	271.061	5.858	4',5,8-Trihydroxyflavanone		HMDB0031824	Phenylpropanoids and polyketides	Flavonoids	C15H12O5	-0.209
3.23_237.1233m/z	237.123	3.226	Nigellimine N-oxide		HMDB0033436	Organic acids and derivatives	Carboxylic acids and derivatives	C12H13NO3	-0.313

3.23_187.0633n	188.071	3.226	Indoleacrylic acid		HMDB0000734	Organoheterocyclic compounds	Indoles and derivatives	C11H9NO2	-0.134
0.87_268.1039m/z	268.104	0.867	Adenosine		HMDB0000050	Nucleosides, nucleotides, and analogues	Purine nucleosides	C10H13N5O4	-0.615
11.91_476.3498n	499.339	11.908	(2alpha,3alpha,5alpha,22R,23R)-2,3,22,23-Tetrahydroxy-25-methylergost-24(28)en-6-one	CC(C1CCC2C1(CCC3C2CC(=O)C4C3(CC(C(C4)O)O)C)C)C(C(C(=C)C(C)(C)C)O)O	HMDB0039443	Lipids and lipid-like molecules	Steroids and steroid derivatives	C29H48O5	-0.848
3.86_647.1580m/z	647.158	3.859	Crosatoside A	CC1C(C(C(C(O1)OC2C(C(C(OC2OC3=C(C=C(C=C3)C4=C(C(=O)C5=C(C=C(C=C5O4)O)O)O)OC)CO)O)O)O)O)O	HMDB0039124	Phenylpropanoids and polyketides	Flavonoids	C28H32O16	-0.351
6.39_446.1212n	469.111	6.389	3,6,7-Trihydroxy-4'-methoxyflavone 7-rhamnoside		HMDB0041455	Phenylpropanoids and polyketides	Flavonoids	C22H22O10	-0.304
2.29_276.1554m/z	276.155	2.289	(2S,2'S)-Pyrosaccharopine		HMDB0038676	Organic acids and derivatives	Carboxylic acids and derivatives	C11H18N2O5	0.011
5.30_400.1517n	401.159	5.301	Italipyrone		HMDB0041307	Benzenoids	Benzene and substituted derivatives	C22H24O7	-1.251
0.83_204.0867m/z	204.087	0.833	N-Acetyl-D-glucosamine		HMDB0000215	Organic oxygen compounds	Organooxygen compounds	C8H15NO6	0.215

3.63_402.1525n	401.145	3.632	Benzyl beta-primeveroside		HMDB0041190	Organic oxygen compounds	Organooxygen compounds	C18H26O10	-0.227
3.49_227.0914m/z	227.091	3.486	threo-Syringoylglycerol		HMDB0031237	Benzenoids	Phenols	C11H16O6	-0.050
0.82_504.1690n	543.132	0.816	6-Kestose	C(C1C(C(C(C(O1)OC2(C(C(C(O2)COC3(C(C(C(O3)CO)O)O)CO)O)O)CO)O)O)O)O	HMDB0033673	Organic oxygen compounds	Organooxygen compounds	C18H32O16	-0.158
6.24_254.1881n	277.177	6.239	Lubiminol		HMDB0029604	Lipids and lipid-like molecules	Prenol lipids	C15H26O3	-0.199
6.86_390.1677n	413.157	6.856	Rosmic acid		HMDB0032082	Lipids and lipid-like molecules	Prenol lipids	C21H26O7	-0.441
0.87_328.1390m/z	328.139	0.867	(E)-2-O-Cinnamoyl-beta-D-glucopyranose		HMDB0035880	Organic oxygen compounds	Organooxygen compounds	C15H18O7	-0.276
3.67_433.2082m/z	433.208	3.674	5a,6a-Epoxy-7E-megastigmene-3a,9e-diol 3-glucoside		HMDB0031676	Organic oxygen compounds	Organooxygen compounds	C19H32O8	0.631
0.85_127.0391m/z	127.039	0.850	5-Hydroxymethyl-2-furancarboxaldehyde		HMDB0034355	Organic oxygen compounds	Organooxygen compounds	C6H6O3	1.142
7.04_181.1222m/z	181.122	7.044	Dihydroactinidiolide		HMDB0035240	Organoheterocyclic compounds	Benzofurans	C11H16O2	-0.440

10.58_572.2959n	595.285	10.581	Ganoderic acid H	CC(CC(=O)CC(C)C(=O)O)C1CC(=O)C2(C1(C(C(=O)C3=C2C(=O)CC4C3(CCC(C4(C)C)O)C)OC(=O)C)C)C	HMDB0035987	Lipids and lipid-like molecules	Prenol lipids	C32H44O9	-4.664
3.00_406.2206n	429.210	2.998	Kiwiionoside	CC(C=CC1(C(CC(CC1(C)O)OC2C(C(C(C(O2)CO)O)O)O)(C)C)O)O	HMDB0038691	Lipids and lipid-like molecules	Prenol lipids	C19H34O9	0.748
6.26_454.1620n	477.152	6.261	KB 2		HMDB0033666	Phenylpropanoids and polyketides	Flavonoids	C25H26O8	-1.773
3.50_393.1768m/z	393.177	3.503	Isopentyl gentiobioside		HMDB0041512	Lipids and lipid-like molecules	Fatty Acyls	C17H32O11	0.526
1.24_164.0473n	182.081	1.243	2-Hydroxycinnamic acid		HMDB0002641	Phenylpropanoids and polyketides	Cinnamic acids and derivatives	C9H8O3	0.022
3.48_357.1192m/z	357.119	3.481	Moringyne		HMDB0031724	Organic oxygen compounds	Organooxygen compounds	C15H20O7	0.310
3.57_418.1476n	417.140	3.567	Oleoside dimethyl ester		HMDB0031350	Lipids and lipid-like molecules	Prenol lipids	C18H26O11	0.236
0.91_156.0655m/z	156.066	0.906	N-Acetyl-L-glutamate 5-semialdehyde		HMDB0006488	Organic acids and derivatives	Carboxylic acids and derivatives	C7H11NO4	-0.076
0.83_188.0917m/z	188.092	0.833	3,4-Dihydroxyphenylglycol		HMDB0000318	Benzenoids	Phenols	C8H10O4	0.060

4.58_562.1678n	563.175	4.575	Chrysin 7-[rhamnosyl-(1->4)-glucoside]	CC1C(C(C(C(O1)OC2C(OC(C(C2O)O)OC3=CC(=C4C(=C3)OC(=CC4=O)C5=CC=CC=C5)O)CO)O)O)O	HMDB0039934	Phenylpropanoids and polyketides	Flavonoids	C27H30O13	-1.482
6.41_301.1069m/z	301.107	6.411	5,7-Dihydroxy-4'-methoxy-8-methylflavanone		HMDB0041321	Phenylpropanoids and polyketides	Flavonoids	C17H16O5	-0.339
3.59_340.1309n	341.138	3.589	Dolichin B		HMDB0029468	Phenylpropanoids and polyketides	Isoflavonoids	C20H20O5	-0.525
4.30_415.1975m/z	415.198	4.298	(3S,7E,9S)-9-Hydroxy-4,7-megastigmadien-3-one 9-glucoside		HMDB0036822	Lipids and lipid-like molecules	Fatty Acyls	C19H30O7	0.392
5.37_266.1517n	289.141	5.365	Arlatin		HMDB0035740	Lipids and lipid-like molecules	Prenol lipids	C15H22O4	-0.532
0.89_324.0358n	323.028	0.894	Uridine 5'-monophosphate		HMDB0000288	Nucleosides, nucleotides, and analogues	Pyrimidine nucleotides	C9H13N2O9P	-0.140
4.49_621.1392m/z	621.139	4.492	Mulberrofuran S	CC12C=C3C(=C(OC4=CC(=CC(=C43)O)C5=CC6=C(O5)C=C(C=C6)O)C7=C(C=C(C=C7)O)O)C(C1O)C8=C(O2)C=C(C=C8)O	HMDB0033985	Phenylpropanoids and polyketides	2-arylbenzofuran flavonoids	C34H24O9	-1.845

4.00_313.0916m/z	313.092	4.004	5-Aminoimidazole ribonucleotide		HMDB0001235	Organic oxygen compounds	Organooxygen compounds	C8H14N3O7P	2.887
3.22_360.1422n	405.140	3.224	Deoxyloganic acid		HMDB0037028	Lipids and lipid-like molecules	Prenol lipids	C16H24O9	0.529
5.62_268.1674n	269.175	5.620	3,11,12-Trihydroxy-1(10)-spirovetiven-2-one		HMDB0038154	Lipids and lipid-like molecules	Prenol lipids	C15H24O4	-0.185
1.18_379.1209m/z	379.121	1.184	4-O-Methylgalactinol		HMDB0033558	Organic oxygen compounds	Organooxygen compounds	C13H24O11	-0.411
4.34_219.1743m/z	219.174	4.342	Curcumol		HMDB0038122	Lipids and lipid-like molecules	Prenol lipids	C15H24O2	-0.129
3.94_370.1416n	371.149	3.941	Dihydrocurcumin		HMDB0031552	Phenylpropanoids and polyketides	Diarylheptanoids	C21H22O6	0.016
4.34_201.1638m/z	201.164	4.342	Rotundone		HMDB0036443	Lipids and lipid-like molecules	Prenol lipids	C15H22O	0.069
1.18_118.0865m/z	118.086	1.184	Angelic acid		HMDB0029608	Lipids and lipid-like molecules	Fatty Acyls	C5H8O2	2.030
13.20_616.4336n	639.424	13.205	Tsugarioside B	CC(=CCCC(COC1C(C(C(CO1)O)O)O)C2CCC3(C2(CCC4=C3CCC5C4(CCC(C5(C)C)OC(=O)C)C)C)C)C	HMDB0035509	Lipids and lipid-like molecules	Prenol lipids	C37H60O7	-0.438

3.86_625.1759m/z	625.176	3.859	Pasternoside	CC1C(C(C(C(O1)OC2=C(C=C(C=C2)C3=C(C(=O)C4=C(C=C(C=C4O3)O)O)OC5C(C(C(O5)C(CO)O)O)O)OC)O)O)O	HMDB0037743	Phenylpropanoids and polyketides	Flavonoids	C28H32O16	-0.708
4.62_501.2343m/z	501.234	4.622	Eriojaposide A	CC1=CC(=O)CC(C1C=CC(C)OC2C(C(C(C(O2)COC3C(C(C(CO3)O)O)O)O)O)O)(C)C	HMDB0038028	Lipids and lipid-like molecules	Fatty Acyls	C24H38O11	0.263
0.82_308.1340m/z	308.134	0.816	Furaneol 4-glucoside		HMDB0032992	Organic oxygen compounds	Organooxygen compounds	C12H18O8	-0.132
3.80_341.1239m/z	341.124	3.803	Coniferin		HMDB0013682	Organic oxygen compounds	Organooxygen compounds	C16H22O8	-0.714
4.06_429.2128m/z	429.213	4.059	Kenposide B	CC(=CCC(COC1C(C(C(C(O1)COC2C(C(C(CO2)O)O)O)O)O)O)C(=C)C)C	HMDB0039749	Lipids and lipid-like molecules	Prenol lipids	C21H36O10	-0.365
1.70_283.1401m/z	283.140	1.704	Agaritinal		HMDB0040694	Organic acids and derivatives	Carboxylic acids and derivatives	C12H15N3O4	-0.074
0.87_130.0500m/z	130.050	0.867	O-Acetylserine		HMDB0003011	Organic acids and derivatives	Carboxylic acids and derivatives	C5H9NO4	0.631
7.34_330.1468n	329.140	7.344	Fragransol B		HMDB0033538	Phenylpropanoids and polyketides	2-arylbenzofuran flavonoids	C19H22O5	0.251

0.80_176.0918m/z	176.092	0.799	2-Aminoheptanedioic acid		HMDB0034252	Organic acids and derivatives	Carboxylic acids and derivatives	$C_7H_{13}NO_4$	0.124
3.69_339.1050m/z	339.105	3.694	Hydroxytyrosol 1-O-glucoside		HMDB0041024	Organic oxygen compounds	Organooxygen compounds	$C_{14}H_{20}O_8$	0.000
12.40_615.4043m/z	615.404	12.401	Schottenol 3-glucoside	CC(=CCC(COC1C(C(C(C(O1)COC2C(C(C(CO2)O)O)O)O)O)O)C(=C)C)C	HMDB0034185	Lipids and lipid-like molecules	Steroids and steroid derivatives	$C_{35}H_{60}O_6$	3.786
3.63_153.1274m/z	153.127	3.631	(-)-trans-Carveol		HMDB0003450	Lipids and lipid-like molecules	Prenol lipids	$C_{10}H_{16}O$	-0.028
5.83_203.1795m/z	203.180	5.834	alpha-Cyperol		HMDB0035026	Lipids and lipid-like molecules	Prenol lipids	$C_{15}H_{24}O$	0.412
1.42_132.1018m/z	132.102	1.422	L-Isoleucine		HMDB0000172	Organic acids and derivatives	Carboxylic acids and derivatives	$C_6H_{13}NO_2$	-0.577
5.45_754.1769m/z	754.177	5.454	Cyanidin 3-(6-caffeoylglucoside) 5-glucoside	C1=CC(=C(C=C1C=CC(=O)OCC2C(C(C(C(O2)OC3=C([O+]=C4C=C(C=C(C4=C3)OC5C(C(C(C(O5)CO)O)O)O)O)C6=CC(=C(C=C6)O)O)O)O)O)O)O	HMDB0037983	Phenylpropanoids and polyketides	Flavonoids	$C_{36}H_{37}O_{19}^+$	2.406
2.59_126.0320n	127.039	2.586	Maltol		HMDB0030776	Organoheterocyclic compounds	Pyrans	$C_6H_6O_3$	2.716

13.00_711.2396m/z	711.240	12.997	Licorice glycoside E	C1C(OC2=C(C1=O)C=CC(=C2)O)C3=CC=C(C=C3)OC4C(C(C(C(O4)CO)O)O)OC5C(C(CO5)(COC(=O)C6=CNC7=CC=CC=C76)O)O	HMDB0031996	Phenylpropanoids and polyketides	Flavonoids	C35H35NO14	-0.003
10.62_435.2506m/z	435.251	10.624	Boviquinone 4	CC(=CCCC(=CCCC(=CCCC(=CCC1=C(C(=O)C=C(C1=O)O)O)C)C)C)C	HMDB0030057	Lipids and lipid-like molecules	Prenol lipids	C26H36O4	-0.059
4.07_482.2594m/z	482.259	4.067	Linalool 3,7-oxide beta-primeveroside	CC1(C(CCC(O1)(C)C=C)OC2C(C(C(C(O2)COC3C(C(C(CO3)O)O)O)O)O)O)C	HMDB0036571	Organic oxygen compounds	Organooxygen compounds	C21H36O11	-0.316
10.60_349.2735m/z	349.274	10.602	2-Hydroxy-6-pentadecylbenzoic acid	CCCCCCCCCCCCCCCC1=C(C(=CC=C1)O)C(=O)O	HMDB0029683	Benzenoids	Benzene and substituted derivatives	C22H36O3	-0.546
1.18_217.0682m/z	217.068	1.184	Methyl beta-D-glucopyranoside		HMDB0029965	Organic oxygen compounds	Organooxygen compounds	C7H14O6	-0.111
3.82_344.1623n	327.159	3.818	2-(4-Allyl-2-methoxyphenoxy)-1-(4-hydroxy-3-methoxyphenyl)-1-propanol		HMDB0031753	Lignans, neolignans and related compounds	Unclassified	C20H24O5	-0.082

0.71_129.0791n	147.113	0.709	Pipecolic acid		HMDB0000070	Organic acids and derivatives	Carboxylic acids and derivatives	C6H11NO2	0.908
3.67_411.1990m/z	411.199	3.673	Icariside B8		HMDB0036846	Lipids and lipid-like molecules	Prenol lipids	C19H32O8	0.281
1.38_231.0839m/z	231.084	1.385	Ethyl beta-D-glucopyranosid e		HMDB0029968	Organic oxygen compounds	Organooxygen compounds	C8H16O6	-0.096
12.44_622.4675m/z	622.467	12.443	Ginsenoside Rh3	CC(=CCC=C(C)C1CCC2(C1C(C C3C2(CCC4C3(CCC(C4(C)C)OC 5C(C(C(C(O5)CO)O)O)O)C)C)O)C)C	HMDB0039645	Lipids and lipid-like molecules	Prenol lipids	C36H60O7	-0.395
3.95_353.1454m/z	353.145	3.952	Isopropyl apiosylglucoside		HMDB0041513	Organic oxygen compounds	Organooxygen compounds	C14H26O10	0.128
4.17_485.1643m/z	485.164	4.168	Cytidine		HMDB0000089	Nucleosides, nucleotides, and analogues	Pyrimidine nucleosides	C9H13N3O5	1.106
3.23_266.1265n	267.134	3.226	N5-(4-Methoxybenzyl) glutamine		HMDB0033598	Organic acids and derivatives	Carboxylic acids and derivatives	C13H18N2O4	-0.665
1.45_260.0897n	305.088	1.454	3-Furanmethanol glucoside		HMDB0032924	Organic oxygen compounds	Organooxygen compounds	C11H16O7	0.445
1.20_324.0359n	305.018	1.197	Pseudouridine 5'-phosphate		HMDB0001271	Organic oxygen compounds	Organooxygen compounds	C9H13N2O9P	0.169
7.67_325.2021m/z	325.202	7.669	Fauronyl acetate		HMDB0036422	Lipids and lipid-like	Prenol lipids	C17H28O3	0.253

					molecules			
4.11_402.1677n	403.175	4.109	Clusin	HMDB0029542	Lignans, neolignans and related compounds	Furanoid lignans	C22H26O7	-0.353
7.51_343.2126m/z	343.213	7.514	Butyl 3-hydroxy-2-methylidenebutanoate	HMDB0040202	Organic acids and derivatives	Hydroxy acids and derivatives	C9H16O3	0.090
0.85_110.0603m/z	110.060	0.850	(S)-2,3,4,5-Tetrahydropiperidine-2-carboxylate	HMDB0012130	Organic acids and derivatives	Carboxylic acids and derivatives	C6H9NO2	2.364
5.39_292.1905m/z	292.191	5.387	[6]-Dehydroshogaol	HMDB0033090	Phenylpropanoids and polyketides	Cinnamic acids and derivatives	C17H22O3	-0.631
3.47_294.1580n	295.165	3.465	Sinapoylputrescine	HMDB0033464	Phenylpropanoids and polyketides	Cinnamic acids and derivatives	C15H22N2O4	0.015
7.67_299.0562m/z	299.056	7.669	5,7-Dihydroxyflavone	HMDB0036619	Phenylpropanoids and polyketides	Flavonoids	C15H10O4	0.187
3.17_298.0969m/z	298.097	3.166	5'-Methylthioadenosine	HMDB0001173	Nucleosides, nucleotides, and analogues	5'-deoxyribonucleosides	C11H15N5O3S	0.171
7.38_346.1416n	369.131	7.384	Alectrol	HMDB0041372	Organoheterocyclic compounds	Lactones	C19H22O6	-0.212
12.42_341.1426m/z	341.143	12.421	Lepidine C	HMDB0032717	Benzenoids	Benzene and substituted derivatives	C21H20N4O2	4.942

3.52_391.1247m/z	391.125	3.524	Di-O-methylcrenatin	HMDB0032742	Organic oxygen compounds	Organooxygen compounds	C15H22O9	0.462
4.32_451.2106m/z	451.211	4.319	Lucidenic acid D1	HMDB0038199	Lipids and lipid-like molecules	Prenol lipids	C27H34O7	-4.223
3.53_185.0809m/z	185.081	3.528	Acetylcholine	HMDB0000895	Organic nitrogen compounds	Organonitrogen compounds	C7H16NO2+	-2.426
7.82_427.1726m/z	427.173	7.821	2-(4-Allyl-2,6-dimethoxyphenoxy)-1-(3-hydroxy-4,5-dimethoxyphenyl)-1-propanol	HMDB0039349	Lignans, neolignans and related compounds	Unclassified	C22H28O7	-0.291
3.17_378.1166m/z	378.117	3.166	Sideritiflavone	HMDB0038356	Phenylpropanoids and polyketides	Flavonoids	C18H16O8	-4.795
3.89_377.1818m/z	377.182	3.888	6Z-8-Hydroxygeraniol 8-O-glucoside	HMDB0035025	Lipids and lipid-like molecules	Prenol lipids	C16H28O7	0.185
2.20_213.0733m/z	213.073	2.201	Diethyl L-malate	HMDB0040220	Organic acids and derivatives	Hydroxy acids and derivatives	C8H14O5	-0.377
3.90_355.1725m/z	355.173	3.901	(2E,4E,7R)-2,7-Dimethyl-2,4-octadiene-1,8-diol 8-O-b-D-glucopyranoside	HMDB0038747	Lipids and lipid-like molecules	Fatty Acyls	C16H28O7	-0.539
2.87_280.1422n	281.150	2.872	Feruloyl-2-hydroxyputrescine	HMDB0033465	Phenylpropanoids and polyketides	Cinnamic acids and derivatives	C14H20N2O4	-0.264

8.77_651.1605m/z	651.161	8.771	3-O-alpha-D-Glucopyranuronosyl-D-xylose		HMDB0039723	Organic oxygen compounds	Organooxygen compounds	C11H18O11	-3.071
11.61_377.1426m/z	377.143	11.608	Glycylglycylglycine		HMDB0029419	Organic acids and derivatives	Carboxylic acids and derivatives	C6H11N3O4	-0.035
6.28_331.1171m/z	331.117	6.282	(S)-5'-Deoxy-5'-(methylsulfinyl)adenosine		HMDB0033662	Nucleosides, nucleotides, and analogues	5'-deoxyribonucleosides	C11H15N5O4S	-3.729
1.13_306.9971m/z	306.997	1.133	(-)-Dioxibrassinin	CSC(=S)NCC1(C2=CC=CC=C2NC1=O)O	HMDB0038634	Organic acids and derivatives	Carboxylic acids and derivatives	C11H12N2O2S2	-0.203
3.93_441.1763m/z	441.176	3.931	Pteroside P	CC1CC2=CC(=C(C(=C2C1=O)C)CCOC3C(C(C(C(O3)CO)O)O)O)CO	HMDB0036608	Organic oxygen compounds	Organooxygen compounds	C20H28O8	-0.809
3.57_488.2714m/z	488.271	3.569	(R)-1-O-[b-D-Glucopyranosyl-(1->6)-b-D-glucopyranoside]-1,3-octanediol		HMDB0032799	Lipids and lipid-like molecules	Fatty Acyls	C20H38O12	2.727
3.31_387.1297m/z	387.130	3.310	Myzodendrone		HMDB0041273	Organic oxygen compounds	Organooxygen compounds	C16H22O8	0.029

3.95_763.2097m/z	763.210	3.952	Kaempferol 3-(2"-rhamnosyl-6"-acetylgalactoside) 7-rhamnoside	CC1C(C(C(C(O1)OC2C(C(C(OC2OC3=C(OC4=CC(=CC(=C4C3=O)O)OC5C(C(C(C(O5)C)O)O)O)C6=CC=C(C=C6)O)COC(=O)C)O)O)O)O)O	HMDB0040541	Phenylpropanoids and polyketides	Flavonoids	C35H42O20	0.770
13.48_636.4206n	619.417	13.480	Fasciculic acid B	CC(CCC(C(C)(C)O)O)C1CCC2(C1(C(CC3=C2CCC4C3(CC(C(C4(C)C)O)OC(=O)CC(C)(CC(=O)O)O)C)O)C)C	HMDB0036438	Lipids and lipid-like molecules	Prenol lipids	C36H60O9	-4.958
5.88_679.4025m/z	679.402	5.876	Glucosyl passiflorate	CC(C)C1(CC(OC1O)C(C)C2CCC3(C2(CCC45C3CCC6C4(C5)C(CC(C6(C)C(=O)OC7C(C(C(C(O7)CO)O)O)O)O)O)C)C)O	HMDB0038141	Lipids and lipid-like molecules	Prenol lipids	C37H60O12	-3.907
3.42_631.2248m/z	631.225	3.417	Vanilloloside		HMDB0032013	Organic oxygen compounds	Organooxygen compounds	C14H20O8	0.724

8.33_676.3665n	699.356	8.331	(S)-Nerolidol 3-O-[a-L-rhamnopyranosyl-(1->4)-a-L-rhamnopyranosyl-(1->6)-b-D-glucopyranoside]	CC1C(C(C(C(O1)OC2C(OC(C(C2O)O)OCC3C(C(C(C(O3)OC(C)(CCC=C(C)CCC=C(C)C)C=C)O)O)O)C)O)O)O	HMDB0040846	Organic oxygen compounds	Organooxygen compounds	C33H56O14	-0.769
3.59_309.0944m/z	309.094	3.589	3-Hydroxy-4,6-heptadiyne-1-yl 1-glucoside		HMDB0038964	Lipids and lipid-like molecules	Fatty Acyls	C13H18O7	-0.287
3.34_432.1628n	455.152	3.342	Benzyl gentiobioside	C1=CC=C(C=C1)COC2C(C(C(C(O2)COC3C(C(C(C(O3)CO)O)O)O)O)O)O	HMDB0041515	Organic oxygen compounds	Organooxygen compounds	C19H28O11	-0.820
9.52_597.3023m/z	597.302	9.517	Ganoderic acid alpha		HMDB0033024	Lipids and lipid-like molecules	Prenol lipids	C32H46O9	-1.835
2.01_310.0897m/z	310.090	2.009	Tetraphyllin B		HMDB0029914	Organic oxygen compounds	Organooxygen compounds	C12H17NO7	-0.225
1.41_360.1280m/z	360.128	1.411	Neosaxitoxin		HMDB0029369	Phenylpropanoids and polyketides	Saxitoxins, gonyautoxins, and derivatives	C10H17N7O5	2.216
1.59_376.1372n	421.135	1.594	Riboflavin		HMDB0000244	Organoheterocyclic compounds	Pteridines and derivatives	C17H20N4O6	-2.889
1.18_192.0270n	215.016	1.184	Citric acid		HMDB0000094	Organic acids and derivatives	Carboxylic acids and derivatives	C6H8O7	0.105

3.80_685.1567m/z	685.157	3.803	Occidentoside	C1C(OC2=CC(=CC(=C2C1=O)O)O)C3=CC=C(C(=C3)OC4=C(C(=C(C(=C4O)C(=O)C=CC5=CC=C(C(=C5)O)O)C6C(C(C(C(O6)CO)O)O)O)O	HMDB0030574	Phenylpropanoids and polyketides	Coumarins and derivatives	C36H32O15	0.630
3.36_360.1056m/z	360.106	3.362	1H-Indol-3-ylacetyl-myo-inositol		HMDB0031182	Organoheterocyclic compounds	Indoles and derivatives	C16H19NO7	0.697
5.19_853.2889m/z	853.289	5.195	Tricrocin	CC(=CC=CC=C(C)C=CC=C(C)C(=O)OC1C(C(C(C(O1)COC2C(C(C(C(O2)CO)O)O)O)O)O)O)C=CC=C(C)C(=O)OC3C(C(C(C(O3)CO)O)O)O	HMDB0002376	Lipids and lipid-like molecules	Prenol lipids	C38H54O19	-0.225
3.82_216.1514n	217.159	3.818	1,2-Dehydro-alpha-cyperone	CC1=C2CC(CCC2(C=CC1=O)C)C(=C)C	HMDB0036589	Lipids and lipid-like molecules	Prenol lipids	C15H20O	-0.152
15.21_1027.5809m/z	1027.581	15.213	Ganoderic acid C1	CC(CC(=O)CC(C)C(=O)O)C1CC(=O)C2(C1(CC(=O)C3=C2C(CC4C3(CCC(=O)C4(C)C)C)O)C)C	HMDB0035627	Lipids and lipid-like molecules	Prenol lipids	C30H42O7	2.058

15.35_124.0871m/z	124.087	15.350	L-Histidinol		HMDB0003431	Organic nitrogen compounds	Organonitrogen compounds	C6H11N3O	0.937
3.82_266.1153n	249.112	3.818	3',4',5'-Trimethoxycinnamyl alcohol acetate		HMDB0040891	Benzenoids	Phenol ethers	C14H18O5	-0.606
4.68_697.4129m/z	697.413	4.681	Momordicoside E	CC(C=O)C1CCC2(C1(CCC3(C2CC=C4C3CCC(C4(C)C)OC5C(C(C(C(O5)COC6C(C(C(C(O6)CO)O)O)O)O)O)O)C)C)C	HMDB0035697	Lipids and lipid-like molecules	Steroids and steroid derivatives	C37H60O12	-4.078
4.05_586.1296n	587.137	4.046	Prunin 6''-O-gallate		HMDB0037582	Phenylpropanoids and polyketides	Flavonoids	C28H26O14	-4.477
4.19_365.1202m/z	365.120	4.194	Sphalleroside A		HMDB0032767	Organic oxygen compounds	Organooxygen compounds	C16H22O8	-1.308
3.90_293.1989n	294.206	3.901	Nonivamide		HMDB0029846	Benzenoids	Phenols	C17H27NO3	-0.665
5.50_361.1870m/z	361.187	5.499	(S)-alpha-Terpinyl glucoside		HMDB0029856	Organic oxygen compounds	Organooxygen compounds	C16H28O6	0.530
3.55_380.1682n	379.161	3.546	Prenyl apiosyl-(1->6)-glucoside		HMDB0031956	Lipids and lipid-like molecules	Fatty Acyls	C16H28O10	-0.246
3.51_371.1675m/z	371.168	3.507	Foeniculoside VIII		HMDB0033009	Organic oxygen compounds	Organooxygen compounds	C16H28O8	-0.343

4.60_515.2829m/z	515.283	4.596	Cinncassiol D1 glucoside	CC(COC1C(C(C(C(O1)CO)O)O)O)C2CC3C4(CC5(C2(C3(C(O5)C6C4CCC6(C)O)O)C)O)C	HMDB0034677	Lipids and lipid-like molecules	Prenol lipids	C26H42O10	-4.257
3.98_389.2181m/z	389.218	3.983	5a,6a-Epoxy-7E-megastigmene-3b,9e-diol 9-glucoside		HMDB0038306	Lipids and lipid-like molecules	Fatty Acyls	C19H32O8	2.870
3.25_535.1789m/z	535.179	3.246	(Z)-Resveratrol 3,4'-diglucoside	C1=CC(=CC=C1C=CC2=CC(=CC(=C2)OC3C(C(C(C(O3)CO)O)O)O)O)OC4C(C(C(C(O4)CO)O)O)O	HMDB0039910	Phenylpropanoids and polyketides	Stilbenes	C26H32O13	-3.726
4.17_394.1481n	417.137	4.173	1-(3-Methyl-2-butenoyl)-6-apiosylglucose		HMDB0039952	Lipids and lipid-like molecules	Fatty Acyls	C16H26O11	1.430
7.92_309.2073m/z	309.207	7.925	Nerolidyl acetate		HMDB0039630	Lipids and lipid-like molecules	Prenol lipids	C17H28O2	0.479
3.25_383.1310m/z	383.131	3.246	1-(3,4-Dimethoxyphenyl)-1,2-ethanediol 2-O-b-D-glucoside		HMDB0034627	Organic oxygen compounds	Organooxygen compounds	C16H24O9	-0.672
1.17_117.0548m/z	117.055	1.167	alpha-Ketoisovaleric acid		HMDB0000019	Organic acids and derivatives	Keto acids and derivatives	C5H8O3	1.967
4.28_332.1832n	355.173	4.278	Betulalbuside A		HMDB0035634	Lipids and lipid-like	Fatty Acyls	C16H28O7	-0.839

						molecules			
0.85_124.0394m/z	124.039	0.850	2-Hydroxy-4-imino-2,5-cyclohexadienone		HMDB0031713	Organic nitrogen compounds	Organonitrogen compounds	C6H5NO2	1.002
3.67_386.1932n	409.183	3.673	Citroside A	CC(=O)C=C=C1C(CC(CC1(C)OC2C(C(C(C(O2)CO)O)O)O)O)(C)C	HMDB0030370	Lipids and lipid-like molecules	Prenol lipids	C19H30O8	-2.354
0.77_380.0953m/z	380.095	0.771	Cyclodopa glucoside		HMDB0029833	Organic oxygen compounds	Organooxygen compounds	C15H19NO9	0.215
13.52_462.3342n	485.324	13.522	Dolichosterone	CC(C)C(=C)C(C(C(C)C1CCC2C1(CCC3C2CC(=O)C4C3(CC(C(C4)O)O)C)C)O)O	HMDB0034336	Lipids and lipid-like molecules	Steroids and steroid derivatives	C28H46O5	-0.800
3.23_236.0917m/z	236.092	3.226	4-Methylumbelliferyl acetate		HMDB0032989	Phenylpropanoids and polyketides	Coumarins and derivatives	C12H10O4	-0.093
0.87_330.0592m/z	330.059	0.867	Cyclic AMP		HMDB0000058	Nucleosides, nucleotides, and analogues	Purine nucleotides	C10H12N5O6P	-1.857
1.14_289.0177m/z	289.018	1.136	cis-Resveratrol 3-sulfate		HMDB0041712	Phenylpropanoids and polyketides	Stilbenes	C14H12O6S	0.336
13.15_381.1739m/z	381.174	13.149	S-Japonin		HMDB0035802	Lipids and lipid-like molecules	Prenol lipids	C19H28O3S	-0.646

3.31_407.0947m/z	407.095	3.314	Tomenin	HMDB0033900	Phenylpropanoids and polyketides	Coumarins and derivatives	C17H20O10	-0.467
6.23_307.1188m/z	307.119	6.226	Citrusin C	HMDB0038708	Organic oxygen compounds	Organooxygen compounds	C16H22O7	0.283
4.06_417.2132m/z	417.213	4.059	9-Hydroxy-7-megastigmen-3-one glucoside	HMDB0040701	Lipids and lipid-like molecules	Fatty Acyls	C19H32O7	0.620
3.25_300.1208n	345.119	3.245	2-(3-Hydroxyphenyl)ethanol 1'-glucoside	HMDB0038332	Organic oxygen compounds	Organooxygen compounds	C14H20O7	-0.221
4.43_291.1832n	292.190	4.427	Norcapsaicin	HMDB0036327	Benzenoids	Phenols	C17H25NO3	-0.780
3.76_343.1291m/z	343.129	3.757	Avenanthramide L	HMDB0033193	Benzenoids	Benzene and substituted derivatives	C18H15NO5	0.768
5.13_495.2201m/z	495.220	5.131	(8S,8'S)-Secoisolariciresinol 9-xyloside	HMDB0037084	Lignans, neolignans and related compounds	Lignan glycosides	C25H34O10	-4.729
0.80_539.1383m/z	539.138	0.804	Dihydroprudomenin	HMDB0039357	Phenylpropanoids and polyketides	Flavonoids	C23H26O12	-4.622
10.13_278.1517n	279.159	10.134	Dibutyl phthalate	HMDB0033244	Benzenoids	Benzene and substituted derivatives	C16H22O4	-0.321
1.18_138.0431n	139.050	1.184	Urocanic acid	HMDB0000301	Organoheterocyclic compounds	Azoles	C6H6N2O2	1.459
0.82_138.0550m/z	138.055	0.816	Trigonelline	HMDB0000875	Alkaloids and	Unclassified	C7H7NO2	-0.011

						derivatives			
6.71_290.1880n	291.195	6.709	Octyl 4-methoxycinnamic acid		HMDB0061861	Phenylpropanoids and polyketides	Cinnamic acids and derivatives	C18H26O3	-0.504
3.98_533.1265m/z	533.127	3.983	2'',6''-Diacetylorientin		HMDB0038777	Phenylpropanoids and polyketides	Flavonoids	C25H24O13	-4.595
0.85_422.0828n	423.090	0.850	Trehalose 6-phosphate		HMDB0001124	Organic oxygen compounds	Organooxygen compounds	C12H23O14P	0.531
3.71_592.1771n	575.174	3.714	Isowertin 2''-rhamnoside	CC1C(C(C(C(O1)OC2C(C(C(OC2C3=C(C=C(C4=C3OC(=CC4=O)C5=CC=C(C=C5)O)O)OC)CO)O)O)O)O)O	HMDB0037417	Phenylpropanoids and polyketides	Flavonoids	C28H32O14	-3.510
4.15_306.2061m/z	306.206	4.152	Capsaicin		HMDB0002227	Benzenoids	Phenols	C18H27NO3	-0.807
4.53_450.1731m/z	450.173	4.533	4-Hydroxytamoxifen sulfate		HMDB0061123	Phenylpropanoids and polyketides	Stilbenes	C26H29NO5S	-0.452
4.19_153.0546m/z	153.055	4.194	Phenoxyacetic acid		HMDB0031609	Benzenoids	Benzene and substituted derivatives	C8H8O3	-0.093
1.28_323.0984m/z	323.098	1.283	3-Hydroxymethylglutaric acid		HMDB0000355	Lipids and lipid-like molecules	Fatty Acyls	C6H10O5	0.223
3.32_365.1205m/z	365.121	3.321	Veranisatin A		HMDB0040663	Lipids and lipid-like molecules	Prenol lipids	C16H22O8	-0.407

4.53_499.2518m/z	499.252	4.533	Eriojaposide B	CC1C(C(C(C(O1)OCC2C(C(C(C(O2)OC(C)C=CC3C(=CC(=O)CC3(C)C)C)O)O)O)O)O)O	HMDB0038029	Lipids and lipid-like molecules	Fatty Acyls	C25H40O11	-3.887
0.87_335.0372m/z	335.037	0.867	Cis-Caffeoyl tartaric acid		HMDB0029276	Phenylpropanoids and polyketides	Cinnamic acids and derivatives	C13H12O9	-0.427
0.92_230.9908m/z	230.991	0.916	2-Phospho-D-glyceric acid		HMDB0003391	Organic oxygen compounds	Organooxygen compounds	C3H7O7P	-2.104
3.70_631.1281m/z	631.128	3.696	3,4,5-Trimethoxyphenyl 2,6-digalloylglucoside	COC1=CC(=CC(=C1OC)OC)OC2C(C(C(C(O2)COC(=O)C3=CC(=C(C(=C3)O)O)O)O)O)OC(=O)C4=CC(=C(C(=C4)O)O)O	HMDB0039312	Organic oxygen compounds	Organooxygen compounds	C29H30O17	-3.621
0.01_223.0637m/z	223.064	0.013	Harmalan		HMDB0029834	Alkaloids and derivatives	Harmala alkaloids	C12H12N2	2.637
3.69_495.1472m/z	495.147	3.694	3-(4-Hydroxy-3-methoxyphenyl)-1,2-propanediol 2-O-(galloyl-glucoside)		HMDB0036376	Benzenoids	Benzene and substituted derivatives	C23H28O13	-4.879
3.00_281.0631m/z	281.063	2.998	D-erythro-D-galacto-octitol		HMDB0029953	Organic oxygen compounds	Organooxygen compounds	C8H18O8	-0.930
3.28_258.1618n	281.149	3.282	Flavidulol A		HMDB0039152	Benzenoids	Phenol ethers	C17H22O2	-0.755

7.32_270.0891n	293.078	7.320	Isomedicarpin		HMDB0033305	Phenylpropanoids and polyketides	Isoflavonoids	C16H14O4	-0.356
0.85_395.0948m/z	395.095	0.850	Galactopinitol B		HMDB0035321	Organic oxygen compounds	Organooxygen compounds	C13H24O11	-0.542
4.13_294.2062m/z	294.206	4.131	[8]-Shogaol		HMDB0031463	Benzenoids	Phenols	C17H24O3	-0.643
5.86_279.1930m/z	279.193	5.855	Ipomeatetrahydrofuran		HMDB0040904	Lipids and lipid-like molecules	Prenol lipids	C15H28O3	-0.342
4.25_521.1302m/z	521.130	4.254	Salviaflaside	C1=CC(=C(C=C1CC(C(=O)O)OC(=O)C=CC2=CC(=C(C=C2)O)OC3C(C(C(C(O3)CO)O)O)O)O)O	HMDB0033705	Organic oxygen compounds	Organooxygen compounds	C24H26O13	0.275
0.85_277.0319m/z	277.032	0.850	6-Phosphogluconic acid		HMDB0001316	Organic oxygen compounds	Organooxygen compounds	C6H13O10P	0.111
7.59_327.1201m/z	327.120	7.595	N-Feruloylaspartic acid		HMDB0040830	Organic acids and derivatives	Carboxylic acids and derivatives	C14H15NO7	4.573
13.69_147.0917m/z	147.092	13.692	Quinoline		HMDB0033731	Organoheterocyclic compounds	Quinolines and derivatives	C9H7N	-0.111
0.89_499.1142m/z	499.114	0.886	N,N'-Bis(gamma-glutamyl)cystine		HMDB0038458	Organic acids and derivatives	Carboxylic acids and derivatives	C16H26N4O10S2	-4.241
1.43_176.0658m/z	176.066	1.433	L-Dihydroorotic acid		HMDB0003349	Organic acids and derivatives	Carboxylic acids and derivatives	C5H6N2O4	-4.755
1.68_172.0369m/z	172.037	1.683	L-2-Amino-5-hydroxypentanoic acid		HMDB0031658	Organic acids and derivatives	Carboxylic acids and derivatives	C5H11NO3	-0.843

1.95_281.0219m/z	281.022	1.951	Moracin M		HMDB0033307	Phenylpropanoids and polyketides	2-arylbenzofuran flavonoids	C14H10O4	3.497
4.87_442.1474n	465.137	4.873	1-O-E-Cinnamoyl-(6-arabinosylglucose)		HMDB0030294	Phenylpropanoids and polyketides	Cinnamic acids and derivatives	C20H26O11	-0.266
0.81_579.1776m/z	579.178	0.810	N-gamma-Glutamyl-S-allylcysteine		HMDB0031874	Organic acids and derivatives	Carboxylic acids and derivatives	C11H18N2O5S	-4.180
8.33_823.4598m/z	823.460	8.331	Ginsenoside Rg3	CC(=CCCC(C)(C1CCC2(C1C(CC3C2(CCC4C3(CCC(C4(C)C)OC5C(C(C(C(O5)CO)O)O)OC6C(C(C(C(O6)CO)O)O)O)C)C)O)C)O)C	HMDB0039546	Lipids and lipid-like molecules	Prenol lipids	C42H72O13	-0.844
6.15_508.2287n	509.236	6.154	Gibberellin A37 glucosyl ester	CC12C(CCC3(C1C(C45C3CCC(C4)C(=C)C5)C(=O)OC6C(C(C(C(O6)CO)O)O)O)COC2=O)O	HMDB0038611	Lipids and lipid-like molecules	Prenol lipids	C26H36O10	-4.266
2.15_367.0647m/z	367.065	2.154	L-gamma-Glutamyl-S-allylthio-L-cysteine		HMDB0038515	Organic acids and derivatives	Carboxylic acids and derivatives	C11H18N2O5S2	2.572
3.34_441.1003m/z	441.100	3.342	Gluconasturtiin		HMDB0038423	Organic oxygen compounds	Organooxygen compounds	C15H21NO9S2	1.744

1.09_675.0970m/z	675.097	1.089	Prebetanin	C1C(NC(=CC1=CC=[N+]2C(CC3=CC(=C(C=C32)O)OC4C(C(C(C(O4)COS(=O)(=O)[O-])O)O)O)C(=O)O)C(=O)O)C(=O)O	HMDB0029411	Alkaloids and derivatives	Betalains	C24H26N2O16S	-2.351
9.55_557.2733m/z	557.273	9.555	Ganoderenic acid D	CC(CC(=O)C=C(C)C1CC(=O)C2(C1(CC(=O)C3=C2C(CC4C3(CCC(=O)C4(C)C)C)O)C)C)C(=O)O	HMDB0036059	Lipids and lipid-like molecules	Prenol lipids	C30H40O7	-4.571
4.93_629.2037m/z	629.204	4.926	(1R*,3R*,3'S*)-1,2,3,4-Tetrahydro-1-(2-thio-3-pyrrolidinyl)-beta-carboline-3-carboxylic acid		HMDB0034767	Alkaloids and derivatives	Harmala alkaloids	C16H17N3O2S	4.315
0.81_439.0861m/z	439.086	0.810	3,5-Dihydroxyphenyl 1-O-(6-O-galloyl-beta-D-glucopyranoside)		HMDB0039307	Organic oxygen compounds	Organooxygen compounds	C19H20O12	-4.816
1.00_595.0896m/z	595.090	1.000	3,8-Dihydroxy-9-methoxycoumestan		HMDB0030562	Phenylpropanoids and polyketides	Isoflavonoids	C16H10O6	2.362
1.18_329.0842m/z	329.084	1.184	N2-(3-Hydroxysuccinoyl)arginine		HMDB0032765	Organic acids and derivatives	Carboxylic acids and derivatives	C10H18N4O6	-5.517

10.59_501.2237m/z	501.224	10.586	Purothionin AII	CCCCC1C(C(OC(=O)C(C(OC1=O)C)NC(=O)C2=C(C(=CC=C2)NC=O)O)C)OC(=O)CC(C)C	HMDB0039001	Benzenoids	Benzene and substituted derivatives	C26H36N2O9	-0.999
0.85_363.0687m/z	363.069	0.850	Aesculin		HMDB0030820	Phenylpropanoids and polyketides	Coumarins and derivatives	C15H16O9	0.125
0.82_696.2241m/z	696.224	0.816	alpha-Viniferin	C1=CC(=CC=C1C2C3C4=C5C(C(OC5=CC(=C4)O)C6=CC=C(C=C6)O)C7=C8C(C(OC8=CC(=C7)O)C9=CC=C(C=C9)O)C1=C3C(=CC(=C1)O)O2)O	HMDB0030603	Phenylpropanoids and polyketides	2-arylbenzofuran flavonoids	C42H30O9	1.865
1.06_521.1088m/z	521.109	1.064	Isomelitric acid A		HMDB0039523	Benzenoids	Benzene and substituted derivatives	C27H22O12	1.819
15.31_122.0966m/z	122.097	15.308	N,N-Dimethylaniline		HMDB0001020	Organic nitrogen compounds	Organonitrogen compounds	C8H11N	1.690
4.32_328.1155n	351.105	4.321	Ethylvanillin glucoside		HMDB0037682	Organic oxygen compounds	Organooxygen compounds	C15H20O8	-0.929
5.22_443.1681m/z	443.168	5.216	Pisumionoside	CC(=O)C=CC1(C(CC(CC1(C)O)OC2C(C(C(C(O2)CO)O)O)O)(C)C)O	HMDB0039947	Lipids and lipid-like molecules	Prenol lipids	C19H32O9	0.688

8.56_769.3588m/z	769.359	8.565	N1,N5,N10,N14-Tetra-trans-p-coumaroylspermine	C1=CC(=CC=C1C=CC(=O)NCCCN(CCCCN(CCCNC(=O)C=CC2=CC=C(C=C2)O)C(=O)C=CC3=CC=C(C=C3)O)C(=O)C=CC4=CC=C(C=C4)O)O	HMDB0039963	Phenylpropanoids and polyketides	Cinnamic acids and derivatives	C46H50N4O8	-1.042
3.88_432.1271n	455.116	3.880	Licoagroside B		HMDB0036523	Lipids and lipid-like molecules	Saccharolipids	C18H24O12	0.747
0.82_481.2139m/z	481.214	0.816	N2-Maltulosylarginine	C1C(C(C(C(O1)(CNC(CCCN=C(N)N)C(=O)O)O)O)O)OC2C(C(C(C(O2)CO)O)O)O	HMDB0041542	Lipids and lipid-like molecules	Saccharolipids	C18H34N4O12	-0.253
3.55_649.2674m/z	649.267	3.548	Isoliensinine		HMDB0033749	Organoheterocyclic compounds	Isoquinolines and derivatives	C37H42N2O6	-0.002
0.83_312.0991n	351.069	0.833	3',4',5'-Trimethoxyflavone		HMDB0033639	Phenylpropanoids and polyketides	Flavonoids	C18H16O5	-2.132
0.80_541.1352m/z	541.135	0.804	Resveratrol 4'-(6-galloylglucoside)		HMDB0034687	Phenylpropanoids and polyketides	Stilbenes	C27H26O12	0.020
6.17_443.2288m/z	443.229	6.172	Neryl rhamnosyl-glucoside	CC1C(C(C(C(O1)OCC2C(C(C(C(O2)OCC=C(C)CCC=C(C)C)O)O)O)O)O)O	HMDB0029349	Lipids and lipid-like molecules	Prenol lipids	C22H38O10	0.221

1.04_959.1816m/z	959.182	1.044	Myricetin 3-galactoside		HMDB0034358	Phenylpropanoids and polyketides	Flavonoids	C21H20O13	8.408
0.89_174.0519n	173.045	0.885	2-Isopropyl-3-oxosuccinate		HMDB0012149	Organic acids and derivatives	Keto acids and derivatives	C7H10O5	-5.073
3.51_140.0706m/z	140.071	3.507	Benzoic acid		HMDB0001870	Benzenoids	Benzene and substituted derivatives	C7H6O2	0.023
3.55_665.1685m/z	665.169	3.548	8-Hydroxyhesperetin 7-[6-acetylglucosyl-(1->2)-glucoside]	CC(=O)OCC1C(C(C(C(O1)OC2C(C(C(OC2OC3=C(C4=C(C(=C3)O)C(=O)C=C(O4)C5=CC(=C(C=C5)OC)O)O)CO)O)O)O)O)O	HMDB0041232	Phenylpropanoids and polyketides	Flavonoids	C30H34O18	-3.935
1.18_331.1003m/z	331.100	1.184	Phosphoribosylformylglycineamidine		HMDB0000999	Organic oxygen compounds	Organooxygen compounds	C8H16N3O8P	-3.363
4.36_474.1737n	497.163	4.363	6-Feruloylglucose 2,3,4-trihydroxy-3-methylbutylglycoside		HMDB0036214	Lipids and lipid-like molecules	Fatty Acyls	C21H30O12	0.008
2.18_287.1101m/z	287.110	2.183	Salicin		HMDB0003546	Organic oxygen compounds	Organooxygen compounds	C13H18O7	-8.619
0.87_377.0842m/z	377.084	0.867	Chlorogenic acid		HMDB0003164	Organic oxygen compounds	Organooxygen compounds	C16H18O9	-0.328

9.40_555.2925m/z	555.292	9.396	Ganoderic acid eta	CC(CC(C=C(C)C(=O)O)O)C1CC(=O)C2(C1(C(C(=O)C3=C2C(CC4C3(CCC(C4(C)C)O)C)O)O)C)C	HMDB0036309	Lipids and lipid-like molecules	Prenol lipids	C30H44O8	-0.641
4.28_793.1946m/z	793.195	4.278	Raphanusol A	COC1=CC(=CC(=C1O)OC)C=CC(=O)OC2C(OC(C(C2O)O)OC(=O)C=CC3=CC(=C(C(=C3)OC)O)OC)COC4C(C(C(C(O4)CO)O)O)O	HMDB0039175	Phenylpropanoids and polyketides	Cinnamic acids and derivatives	C34H42O19	-0.809
3.96_357.1881m/z	357.188	3.962	3,7-Dimethyl-5-octene-1,7-diol 1-glucoside		HMDB0034771	Lipids and lipid-like molecules	Fatty Acyls	C16H30O7	-0.826
5.90_269.0456m/z	269.046	5.899	2'-Hydroxydaidzein		HMDB0029372	Phenylpropanoids and polyketides	Isoflavonoids	C15H10O5	0.091
2.59_249.1267m/z	249.127	2.586	Dienestrol		HMDB0015027	Phenylpropanoids and polyketides	Stilbenes	C18H18O2	-2.510

4.32_741.2029m/z	741.203	4.321	6-Glucopyranosylprocyanidin B2	C1C(C(OC2=C1C(=CC(=C2C3C(C(OC4=C3C(=C(C(=C4)O)C5C(C(C(C(O5)CO)O)O)O)O)C6=CC(=C(C=C6)O)O)O)O)C7=CC(=C(C=C7)O)O)O	HMDB0037403	Phenylpropanoids and polyketides	Flavonoids	C36H36O17	0.513
1.18_305.0841m/z	305.084	1.184	Xylobiose		HMDB0029894	Organic oxygen compounds	Organooxygen compounds	C10H18O9	-0.579
15.26_226.1590m/z	226.159	15.264	1,2-Diphenylcyclobutane		HMDB0031821	Phenylpropanoids and polyketides	Stilbenes	C16H16	-0.075
0.80_705.1854m/z	705.185	0.799	Mannan	C(C1C(C(C(C(O1)OC2C(OC(C(C2O)O)OC3C(OC(C(C3O)O)OC4C(OC(C(C4O)O)O)CO)CO)CO)O)O)O)O	HMDB0029931	Organic oxygen compounds	Organooxygen compounds	C24H42O21	0.590
3.55_637.1750m/z	637.175	3.548	Catechin 3',5-diglucoside		HMDB0037951	Phenylpropanoids and polyketides	Flavonoids	C27H34O16	1.817
9.03_583.4143m/z	583.414	9.033	Neoxanthin	CC(=CC=CC=C(C)C=CC=C(C)C=C=C1C(CC(CC1(C)O)O)(C)C)C=CC=C(C)C=CC23C(CC(CC2(O3)C)O)(C)C	HMDB0003020	Lipids and lipid-like molecules	Prenol lipids	C40H56O4	-0.412

1.18_393.1635n	416.155	1.184	6'-Apiosyllotaustralin		HMDB0034207	Organic oxygen compounds	Organooxygen compounds	C16H27NO10	-0.061
3.67_633.1422m/z	633.142	3.673	Luteolin 6-C-glucoside 8-C-arabinoside	C1=CC(=C(C=C1C2=CC(=O)C3=C(C(=C(C(=C3O2)C4C(C(C(C(O4)CO)O)O)O)O)C5C(C(C(C(O5)CO)O)O)O)O)O)O	HMDB0029258	Phenylpropanoids and polyketides	Flavonoids	C27H30O16	-0.692
9.39_279.2328m/z	279.233	9.386	(5Z,8Z)-1,5,8-Heptadecatriene		HMDB0041082	Hydrocarbons	Unsaturated hydrocarbons	C17H30	-0.583
12.01_435.2522m/z	435.252	12.012	(R)-Pterosin B		HMDB0030759	Benzenoids	Indanes	C14H18O2	-4.267
1.00_1013.3159m/z	1013.316	1.004	Maltohexaose	C(C1C(C(C(C(O1)OC2C(OC(C(C2O)O)OC3C(OC(C(C3O)O)OC4C(OC(C(C4O)O)OC5C(OC(C(C5O)O)OC6C(OC(C(C6O)O)O)CO)CO)CO)CO)CO)O)O)O)O	HMDB0012253	Organic oxygen compounds	Organooxygen compounds	C36H62O31	-0.809
0.87_291.0475m/z	291.047	0.867	D-Sedoheptulose 7-phosphate		HMDB0001068	Organic oxygen compounds	Organooxygen compounds	C7H15O10P	-0.270
3.84_577.2259m/z	577.226	3.839	Ssioriside	COC1=CC(=CC(=C1O)OC)CC(CO)C(CC2=CC(=C(C(=C2)OC)O)OC)COC3C(C(C(CO3)O)O)O	HMDB0038934	Lignans, neolignans and related compounds	Lignan glycosides	C27H38O12	0.578

0.91_140.0107m/z	140.011	0.906	(S)-2-Azetidinecarboxylic acid		HMDB0029615	Organic acids and derivatives	Carboxylic acids and derivatives	C4H7NO2	-1.324
10.37_517.3498m/z	517.350	10.367	Pokeberrygenin	CC1(C2CCC3(C(C2(CC(C1O)O)C)CC=C4C3(CCC5(C4CC(CC5)(C)C(=O)OC)C(=O)O)C)C)C	HMDB0034653	Lipids and lipid-like molecules	Prenol lipids	C31H48O6	-4.875
3.86_249.1484m/z	249.148	3.859	4-Epiisoinuviscolide		HMDB0031378	Lipids and lipid-like molecules	Prenol lipids	C15H20O3	-0.635
0.87_375.0686m/z	375.069	0.867	Chlorogenoquinone		HMDB0029383	Organic oxygen compounds	Organooxygen compounds	C16H16O9	-0.081

Table S2 248 target proteins from *D. officinale*

Number	Target proteins	Common name
1	Cyclin-dependent kinase 1	CDK1
2	Basic fibroblast growth factor	FGF2
3	Vascular endothelial growth factor A	VEGFA
4	Acidic fibroblast growth factor	FGF1
5	Heparanase	HPSE
6	Galectin-4	LGALS4
7	Galectin-8	LGALS8
8	Gamma-secretase	PSEN2
9	Multidrug resistance-associated protein 1	ABCC1
10	Estradiol 17-beta-dehydrogenase 1	HSD17B1

11	Carbonic anhydrase XII	CA12
12	Testis-specific androgen-binding protein	SHBG
13	Cytochrome P450 19A1	CYP19A1
14	Carbonic anhydrase IV	CA4
15	Cytochrome P450 1B1	CYP1B1
16	Carbonyl reductase [NADPH] 1	CBR1
17	Estrogen receptor alpha	ESR1
18	Estrogen receptor beta	ESR2
19	Cyclooxygenase-1	PTGS1
20	Monoamine oxidase B	MAOB
21	Adenosine A1 receptor	ADORA1
22	Adenosine A3 receptor	ADORA3
23	Prostanoid EP1 receptor	PTGER1
24	Prostanoid EP4 receptor	PTGER4
25	Prostanoid FP receptor	PTGFR
26	Prostanoid EP3 receptor	PTGER3
27	Prostanoid EP2 receptor	PTGER2
28	Prostanoid IP receptor	PTGIR
29	Peroxisome proliferator-activated receptor gamma	PPARG
30	Peroxisome proliferator-activated receptor alpha	PPARA
31	Peroxisome proliferator-activated receptor delta	PPARD
32	Free fatty acid receptor 1	FFAR1
33	Cannabinoid receptor 1	CNR1
34	Sphingosine kinase 1	SPHK1
35	Carbonic anhydrase II	CA2
36	Carbonic anhydrase IX	CA9

37	Carbonic anhydrase VI	CA6
38	Carbonic anhydrase I	CA1
39	Carbonic anhydrase VA	CA5A
40	TNF-alpha	TNF
41	Interleukin-2	IL2
42	Aldose reductase	AKR1B1
43	Xanthine dehydrogenase	XDH
44	ATP-citrate synthase	ACLY
45	Glucocorticoid receptor	NR3C1
46	Androgen Receptor	AR
47	Corticosteroid binding globulin	SERPINA6
48	Sigma opioid receptor	SIGMAR1
49	Dopamine transporter	SLC6A3
50	MAP kinase ERK1	MAPK3
51	Progesterone receptor	PGR
52	11-beta-hydroxysteroid dehydrogenase 2	HSD11B2
53	11-beta-hydroxysteroid dehydrogenase 1	HSD11B1
54	Cytochrome P450 17A1	CYP17A1
55	Niemann-Pick C1-like protein 1	NPC1L1
56	Mineralocorticoid receptor	NR3C2
57	Protein-tyrosine phosphatase 2C	PTPN11
58	Aldo-keto reductase family 1 member B10	AKR1B10
59	Prostaglandin E synthase	PTGES
60	Protein kinase C eta	PRKCH
61	T-cell protein-tyrosine phosphatase	PTPN2
62	LXR-alpha	NR1H3

63	Neprilysin (by homology)	MME
64	Tryptophan 2，3-dioxygenase (by homology)	TDO2
65	Adenosine A2a receptor	ADORA2A
66	Dipeptidyl peptidase IV	DPP4
67	Adenosine kinase	ADK
68	Heat shock cognate 71 kDa protein	HSPA8
69	78 kDa glucose-regulated protein	HSPA5
70	Adenosine deaminase	ADA
71	Adenosylhomocysteinase	AHCY
72	Glyceraldehyde-3-phosphate dehydrogenase liver	GAPDH
73	Histone-lysine N-methyltransferase，H3 lysine-9 specific 5	EHMT1
74	Histone-lysine N-methyltransferase，H3 lysine-9 specific 3	EHMT2
75	Induced myeloid leukemia cell differentiation protein Mcl-1	MCL1
76	Histone-lysine N-methyltransferase SETD7	SETD7
77	Norepinephrine transporter	SLC6A2
78	Somatostatin receptor 5	SSTR5
79	Somatostatin receptor 2	SSTR2
80	Somatostatin receptor 4	SSTR4
81	Somatostatin receptor 1	SSTR1
82	Somatostatin receptor 3	SSTR3
83	UDP-glucuronosyltransferase 2B7	UGT2B7
84	Potassium-transporting ATPase alpha chain 2	ATP12A
85	Acetylcholinesterase	ACHE
86	Butyrylcholinesterase	BCHE

87	Nuclear factor NF-kappa-B p65 subunit	RELA
88	Galectin-3	LGALS3
89	Heat shock protein HSP 90-alpha	HSP90AA1
90	Carbonic anhydrase III	CA3
91	Macrophage migration inhibitory factor	MIF
92	Angiotensin-converting enzyme	ACE
93	Renin	REN
94	Protein phosphatase 2C beta	PPM1B
95	Protein-tyrosine phosphatase 1B	PTPN1
96	Integrin alpha-Ilb	ITGA2B
97	Aldose reductase (by homology)	AKR1A1
98	Ribosomal protein S6 kinase alpha 3	RPS6KA3
99	ATP-binding cassette sub-family G member 2	ABCG2
100	Thymidylate synthase	TYMS
101	Hypoxanthine-guanine phosphoribosyltransferase	HPRT1
102	Aldo-keto-reductase family 1 member C3	AKR1C3
103	Arachidonate 5-lipoxygenase	ALOX5
104	Monoamine oxidase A	MAOA
105	Beta amyloid A4 protein	APP
106	Histone acetyltransferase p300	EP300
107	Toll-like receptor (TLR7/TLR9)	TLR9
108	Adrenergic receptor alpha-2	ADRA2C
109	Alpha-2a adrenergic receptor	ADRA2A
110	NADPH oxidase 4	NOX4
111	Quinone reductase 2	NQO2
112	Metabotropic glutamate receptor 5	GRM5

113	Leukotriene A4 hydrolase	LTA4H
114	Glutamate receptor ionotropic kainate 1	GRIK1
115	Glutamate receptor ionotropic kainate 2	GRIK2
116	Metabotropic glutamate receptor 2	GRM2
117	Plasminogen	PLG
118	Xaa-Pro dipeptidase	PEPD
119	Metabotropic glutamate receptor 3	GRM3
120	Metabotropic glutamate receptor 6	GRM6
121	Excitatory amino acid transporter 2	SLC1A2
122	Metabotropic glutamate receptor 1	GRM1
123	Voltage-gated calcium channel alpha2/delta subunit 1 (by homology)	CACNA2D1
124	Aminopeptidase A	ENPEP
125	Metabotropic glutamate receptor 8	GRM8
126	Histone acetyltransferase PCAF	KAT2B
127	Cytidine deaminase	CDA
128	Dual specificity phosphatase Cdc25A	CDC25A
129	Matrix metalloproteinase 9	MMP9
130	Cyclin-dependent kinase 5/CDK5 activator 1	CDK5R1
131	Cytochrome P450 11B1	CYP11B1
132	Cytochrome P450 11B2	CYP11B2
133	Sulfonylurea receptor 2	ABCC9
134	Sulfonylurea receptor 2， Kir6.2	KCNJ11
135	Matrix metalloproteinase 2	MMP2
136	Neuronal acetylcholine receptor； alpha3	CHRNA3
137	Muscarinic acetylcholine receptor M1	CHRM1

续附表 2

编号	靶点蛋白	Common name
138	Acyl coenzyme A：cholesterol acyltransferase	CES1
139	Serotonin 1a (5-HT1a) receptor	HTR1A
140	Cyclin-dependent kinase 1/cyclin B	CCNB3
141	Cyclin-dependent kinase 6	CDK6
142	P-glycoprotein 1	ABCB1
143	Tankyrase-2	TNKS2
144	Tankyrase-1	TNKS
145	Arachidonate 15-lipoxygenase	ALOX15
146	Arachidonate 12-lipoxygenase	ALOX12
147	Poly [ADP-ribose] polymerase-1	PARP1
148	Lysine-specific demethylase 4D-like	KDM4E
149	G protein-coupled receptor kinase 6	GRK6
150	Casein kinase II alpha	CSNK2A1
151	Tyrosine-protein kinase receptor FLT3	FLT3
152	Cyclooxygenase-2	PTGS2
153	Tyrosine-protein kinase SYK	SYK
154	Glycogen synthase kinase-3 beta	GSK3B
155	Transthyretin	TTR
156	Cystic fibrosis transmembrane conductance regulator	CFTR
157	Tyrosine-protein kinase LCK	LCK
158	Inhibitor of nuclear factor kappa B kinase beta subunit	IKBKB
159	Neurotrophic tyrosine kinase receptor type 2	NTRK2
160	Serine/threonine-protein kinase PIM1	PIM1
161	Receptor-type tyrosine-protein phosphatase S	PTPRS

162	Epidermal growth factor receptor erbB1	EGFR
163	Estradiol 17-beta-dehydrogenase 2	HSD17B2
164	AMY1C	AMY1A
165	NEDD8-activating enzyme E1 regulatory subunit	NAE1
166	Arginase-1 (by homology)	ARG1
167	6-phosphofructo-2-kinase/fructose-2，6-bisphosphatase 3	PFKFB3
168	Calmodulin	CALM1
169	Nitric oxide synthase， inducible (by homology)	NOS2
170	Muscarinic acetylcholine receptor M4	CHRM4
171	Neuronal acetylcholine receptor； alpha4	CHRNA4
172	Muscarinic acetylcholine receptor M5	CHRM5
173	Muscarinic acetylcholine receptor M2	CHRM2
174	Muscarinic acetylcholine receptor M3	CHRM3
175	Neuronal acetylcholine receptor protein alpha-7 subunit	CHRNA7
176	Mu opioid receptor (by homology)	OPRM1
177	Delta opioid receptor (by homology)	OPRD1
178	Protein kinase C alpha	PRKCA
179	Matrix metalloproteinase 12	MMP12
180	Vanilloid receptor (by homology)	TRPV1
181	Cytochrome P450 1A2	CYP1A2
182	Fructose-1，6-bisphosphatase	FBP1
183	Tyrosine-protein kinase SRC	SRC
184	Bis(5'-adenosyl)-triphosphatase	FHIT
185	Vanilloid receptor	IGF2R
186	HMG-CoA reductase	HMGCR
187	Tyrosyl-DNA phosphodiesterase 1	TDP1

188	Serotonin 2a (5-HT2a) receptor (by homology)	HTR2A
189	Serotonin 2c (5-HT2c) receptor	HTR2C
190	Serotonin 7 (5-HT7) receptor	HTR7
191	Serotonin 6 (5-HT6) receptor	HTR6
192	Serotonin 5a (5-HT5a) receptor	HTR5A
193	Nischarin	NISCH
194	Kinesin-like protein 1	KIF11
195	Metabotropic glutamate receptor 4	GRM4
196	Metabotropic glutamate receptor 7	GRM7
197	Glutamate receptor ionotropic， AMPA 1	GRIA1
198	Glutamate receptor ionotropic， AMPA 4	GRIA4
199	Glutamate receptor ionotropic， AMPA 2	GRIA2
200	Phosphodiesterase 4B	PDE4B
201	Phosphodiesterase 4D	PDE4D
202	NAD-dependent deacetylase sirtuin 1	SIRT1
203	Apoptosis regulator Bcl-2	BCL2
204	MAP kinase ERK2	MAPK1
205	Fatty acid binding protein muscle	FABP3
206	Fatty acid binding protein adipocyte	FABP4
207	Anandamide amidohydrolase	FAAH
208	Telomerase reverse transcriptase	TERT
209	Fatty acid binding protein epidermal	FABP5
210	Fatty acid-binding protein， liver	FABP1
211	Acyl-CoA desaturase	SCD
212	Prolyl endopeptidase	PREP
213	DNA polymerase beta	POLB

214	Dopamine D2 receptor (by homology)	DRD2
215	Protein-tyrosine phosphatase 1C	PTPN6
216	Lymphocyte differentiation antigen CD38	CD38
217	Egl nine homolog 1	EGLN1
218	D-amino-acid oxidase	DAO
219	Matrix metalloproteinase 13	MMP13
220	Leukocyte adhesion glycoprotein LFA-1 alpha	ITGAL
221	Thromboxane-A synthase	TBXAS1
222	Maltase-glucoamylase	MGAM
223	Estrogen-related receptor alpha	ESRRA
224	Estrogen-related receptor beta	ESRRB
225	Aldehyde dehydrogenase	ALDH2
226	Tyrosinase (by homology)	TYR
227	CD81 antigen	CD81
228	Carboxylesterase 2	CES2
229	Dual specificity phosphatase Cdc25B	CDC25B
230	Vitamin D receptor	VDR
231	Bile acid receptor FXR	NR1H4
232	Glucose-6-phosphate 1-dehydrogenase	G6PD
233	G-protein coupled bile acid receptor 1	GPBAR1
234	Estradiol 17-beta-dehydrogenase 3	HSD17B3
235	Beta-1，4-galactosyltransferase 1	B4GALT1
236	Dual specificity mitogen-activated protein kinase 1	MAP2K1
237	Dopamine D4 receptor	DRD4

238	Dopamine D3 receptor	DRD3
239	CDK5 activator 1	CDK5
240	Neuronal acetylcholine receptor； beta2	CHRNB2
241	Neuronal acetylcholine receptor； beta4	CHRNB4
242	Integrin beta-3	ITGB3
243	Gamma-secretase	PSENEN
244	Cyclin B1	CCNB1
245	Gamma-secretase	NCSTN
246	Gamma-secretase	APH1A
247	Cyclin B2	CCNB2
248	Gamma-secretase	PSEN1

Table S3 8 core active components from *D. officinale.*

Number	Core ingredients	Degree	Betweenness Centrality	Closeness Centrality
TPSH213	5,7-Dihydroxyflavone	45	0.071	0.429
TPSH33	Mangiferic acid	15	0.027	0.390
TPSH017	Norartocarpanone	16	0.022	0.395
TPSH004	Naringenin	22	0.020	0.402
TPSH221	Sideritoflavone	14	0.018	0.388
TPSH132	4',5,8-Trihydroxyflavanone	14	0.015	0.388
TPSH239	Benzyl gentiobioside	13	0.006	0.386
TPSH28	Rutin	15	0.006	0.391

Table S4 The binding energy of the core components to the core targets.

Core ingredients	Core targets	PDB ID	Binding energy（kcal/mol）
5,7-Dihydroxyflavone	HSP90AA1	4L8Z	-6.72
	SRC	7NG7	-2.81
	EGFR	8A27	-4.82
	MAPK3	2ZOQ	-4.5
	EP300	5NU5	-3.27
	GAPDH	4WNI	-4.2
	ESR1	7UJO	-3.11
	APP	4PWQ	-3.1
	RELA	7O59	-2.78
	PPARG	6T1S	-2.65
Mangiferic acid	HSP90AA1	4L8Z	-2.9
Norartocarpanone	HSP90AA1	4L8Z	-4.51
	MAPK3	2ZOQ	-2.04
	EP300	5NU5	-2.2
	GAPDH	4WNI	-2.76
	APP	4PWQ	-1.64
	RELA	7O59	-2.99
Naringenin	HSP90AA1	4L8Z	-3.56
	EGFR	8A27	-1.83
	MAPK3	2ZOQ	-2.74
	EP300	5NU5	-2.49
	GAPDH	4WNI	-2.73
	ESR1	7UJO	-2.69

	APP	4PWQ	-2.68
	RELA	7O59	-2.17
4',5,8-Trihydroxyflavanone	HSP90AA1	4L8Z	-2.99
	EGFR	8A27	-1.94
	MAPK3	2ZOQ	-2.52
	EP300	5NU5	-2.76
	GAPDH	4WNI	-2.71
	ESR1	7UJO	-1.85
	APP	4PWQ	-2.59
	RELA	7O59	-2.3
	PPARG	6T1S	-1.67
