# Supplementary figures and images for: Mechanistic Elucidation of the Anti-Ageing Effects of Dendrobium officinale via Network Pharmacology and Experimental Validation
Source: Foods. 2025 Oct 3;14(19):3418. doi: 10.3390/foods14193418 (PMC12523731; doi:10.3390/foods14193418)

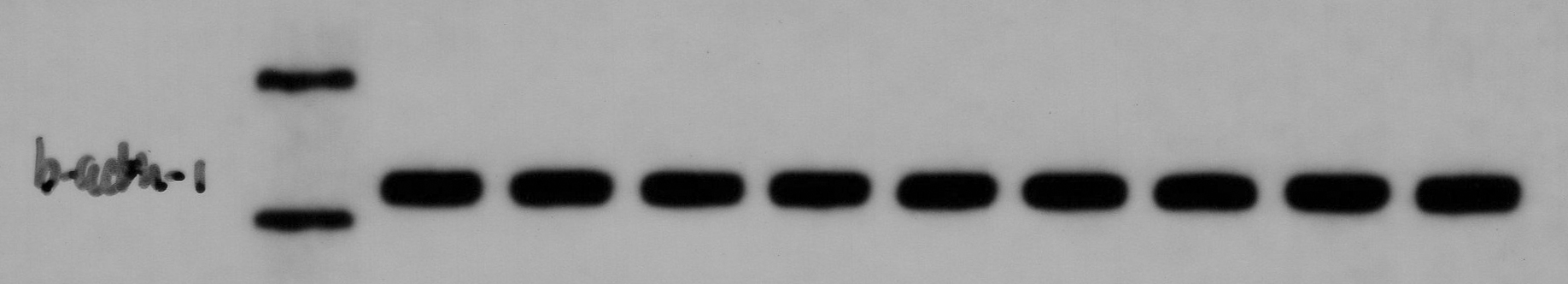

Supplement: Supplementary file 1 [file foods-14-03418-s001.zip › WB Biological replicates/Biological replicates 1/b-actin-1.jpg]

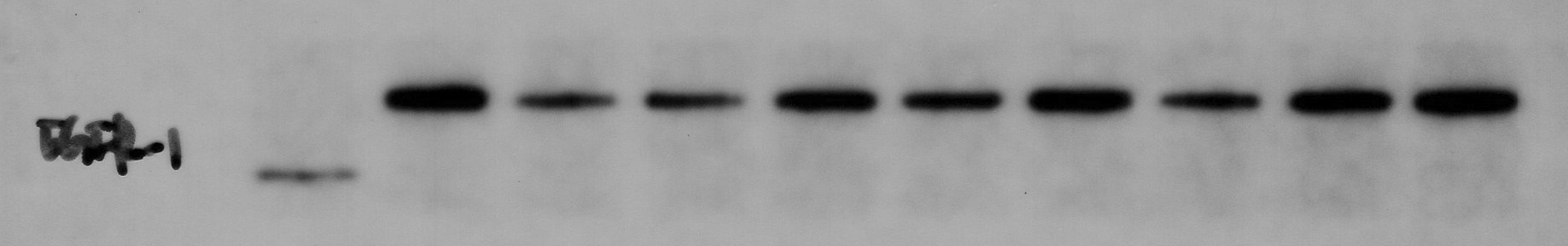

Supplement: Supplementary file 1 [file foods-14-03418-s001.zip › WB Biological replicates/Biological replicates 1/EGFR-1.jpg]

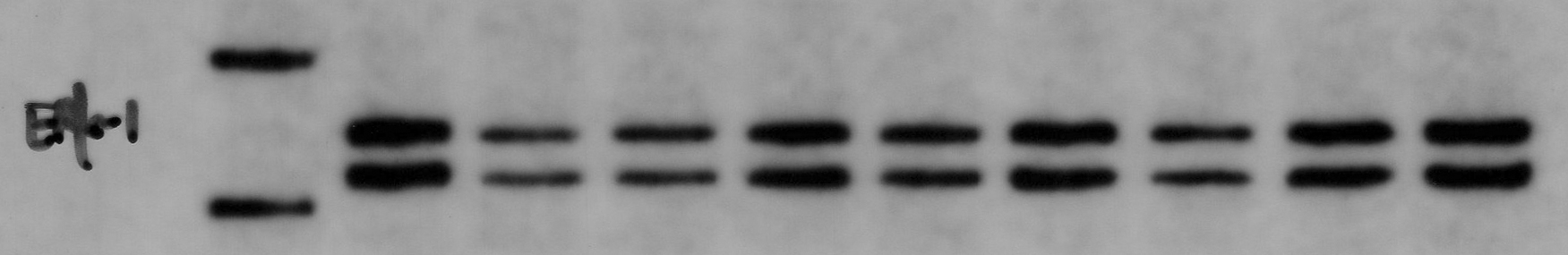

Supplement: Supplementary file 1 [file foods-14-03418-s001.zip › WB Biological replicates/Biological replicates 1/ERK-1.jpg]

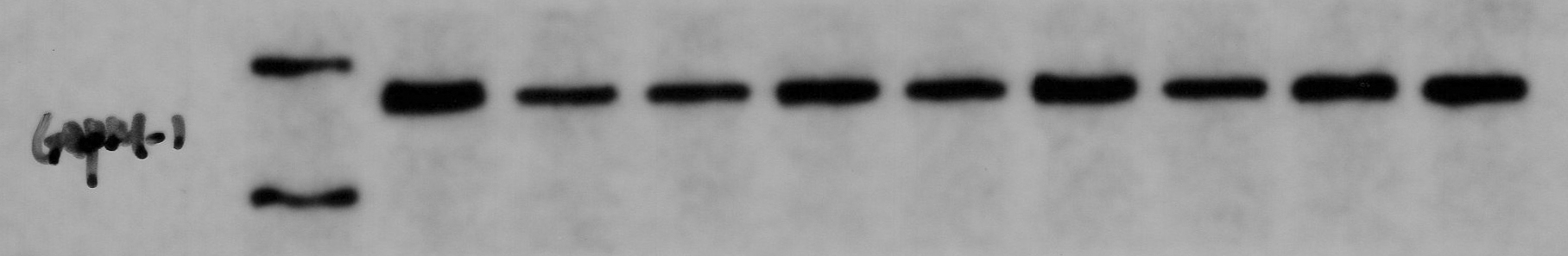

Supplement: Supplementary file 1 [file foods-14-03418-s001.zip › WB Biological replicates/Biological replicates 1/GAPDH-1.jpg]

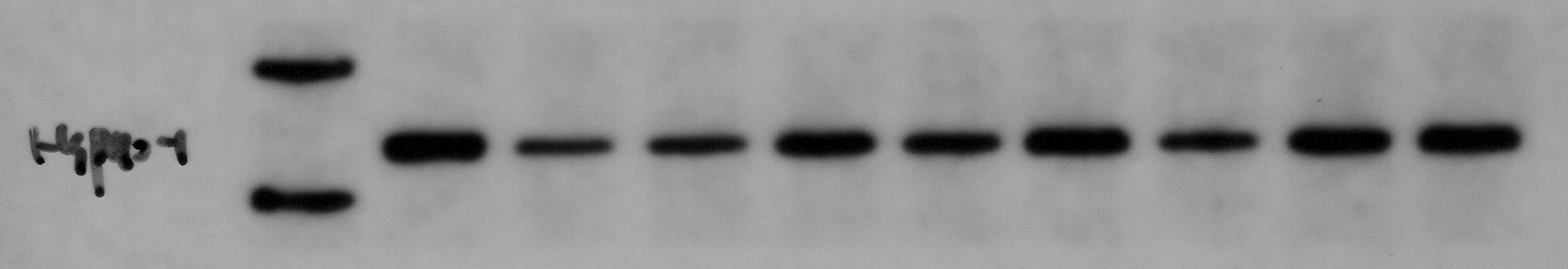

Supplement: Supplementary file 1 [file foods-14-03418-s001.zip › WB Biological replicates/Biological replicates 1/HSP90-1.jpg]

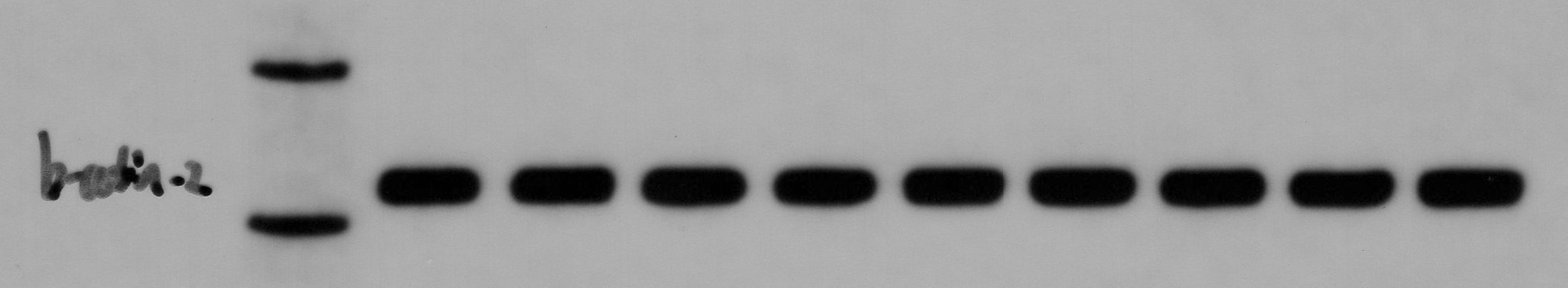

Supplement: Supplementary file 1 [file foods-14-03418-s001.zip › WB Biological replicates/Biological replicates 2/b-actin-2.jpg]

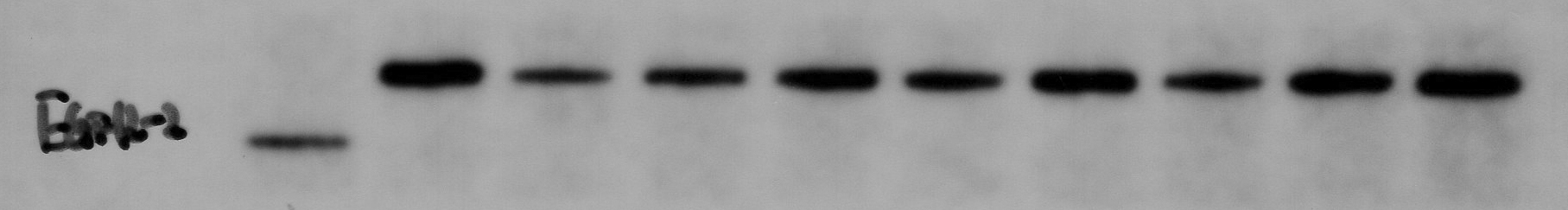

Supplement: Supplementary file 1 [file foods-14-03418-s001.zip › WB Biological replicates/Biological replicates 2/EGFR-2.jpg]

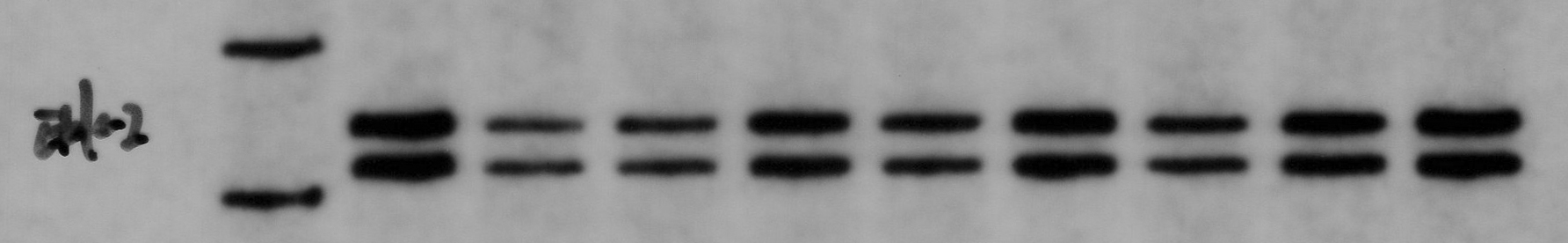

Supplement: Supplementary file 1 [file foods-14-03418-s001.zip › WB Biological replicates/Biological replicates 2/ERK-2.jpg]

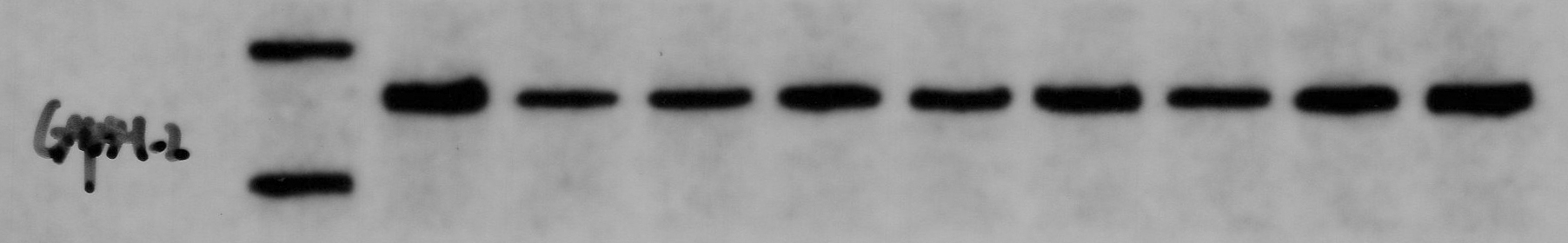

Supplement: Supplementary file 1 [file foods-14-03418-s001.zip › WB Biological replicates/Biological replicates 2/GAPDH-2.jpg]

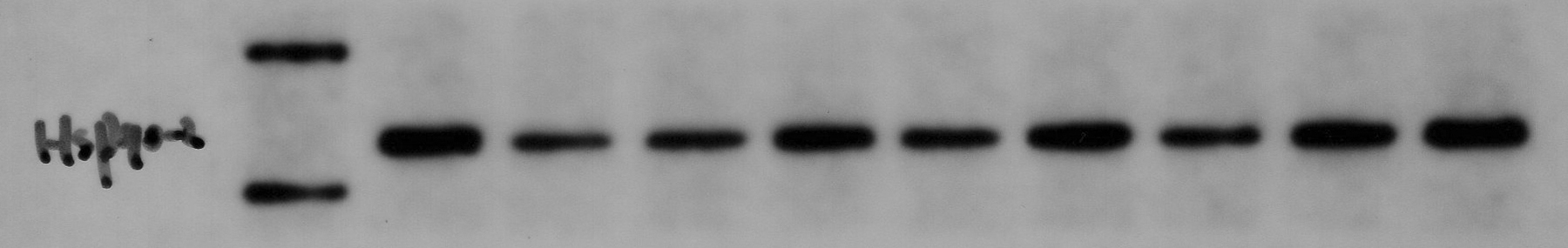

Supplement: Supplementary file 1 [file foods-14-03418-s001.zip › WB Biological replicates/Biological replicates 2/HSP90-2.jpg]

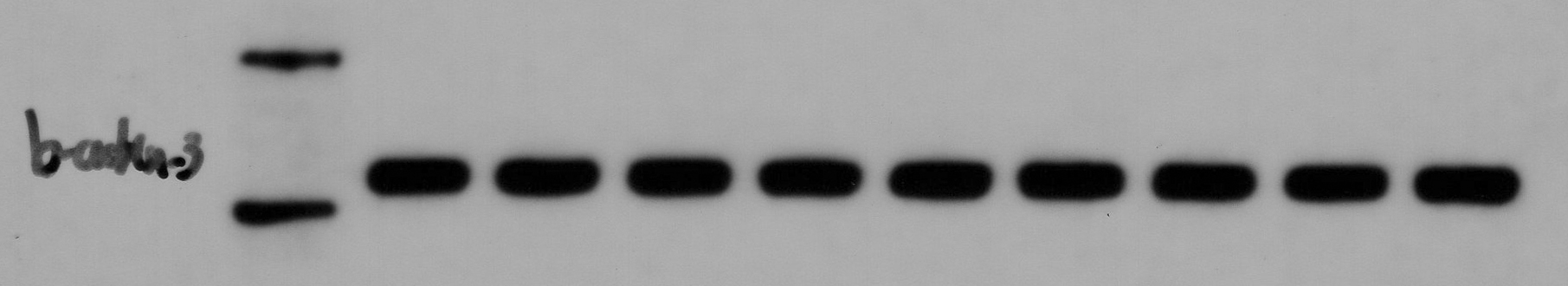

Supplement: Supplementary file 1 [file foods-14-03418-s001.zip › WB Biological replicates/Biological replicates 3/b-actin-3.jpg]

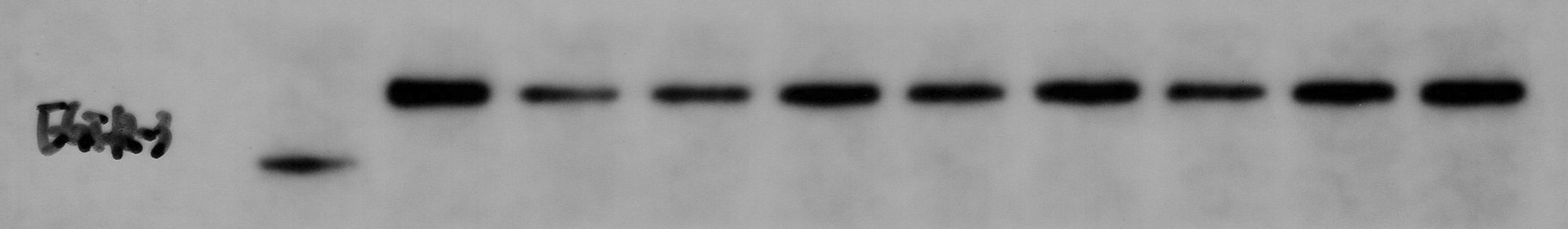

Supplement: Supplementary file 1 [file foods-14-03418-s001.zip › WB Biological replicates/Biological replicates 3/EGFR-3.jpg]

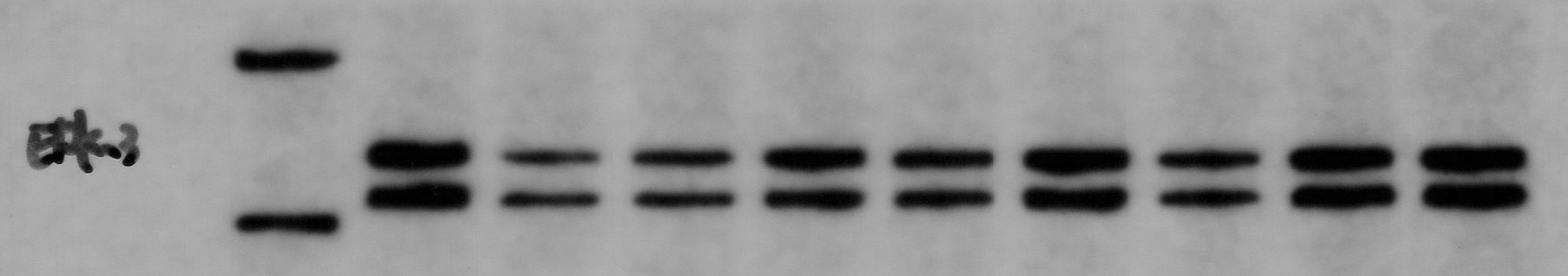

Supplement: Supplementary file 1 [file foods-14-03418-s001.zip › WB Biological replicates/Biological replicates 3/ERK-3.jpg]

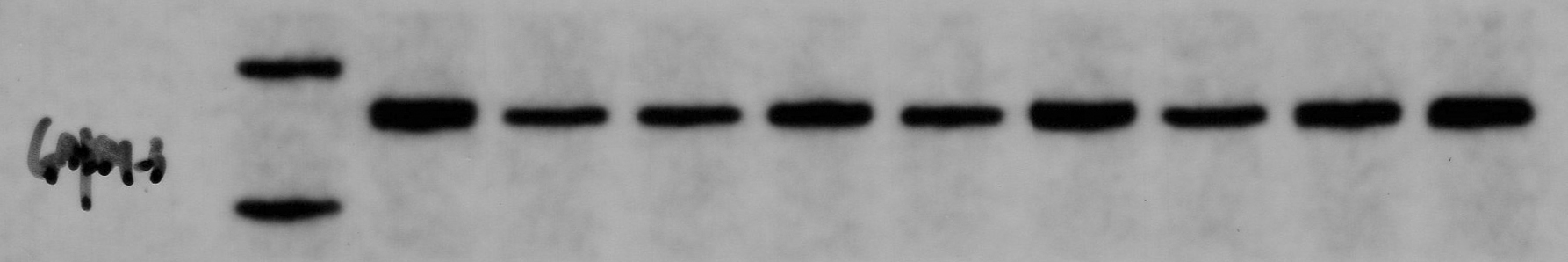

Supplement: Supplementary file 1 [file foods-14-03418-s001.zip › WB Biological replicates/Biological replicates 3/GAPDH-3.jpg]

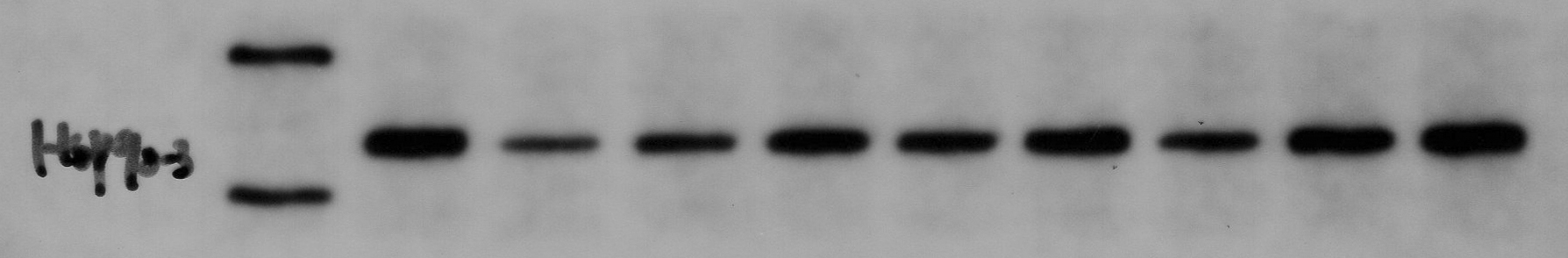

Supplement: Supplementary file 1 [file foods-14-03418-s001.zip › WB Biological replicates/Biological replicates 3/HSP90-3.jpg]
